# Computational design of an improved photoswitchable psychedelic based on light absorption, membrane permeation and protein binding

**DOI:** 10.1039/d5cp01252j

**Published:** 2025-08-08

**Authors:** Vito F. Palmisano, Claudio Agnorelli, Shirin Faraji, Juan J. Nogueira

**Affiliations:** a Institute of Theoretical and Computational Chemistry, Heinrich Heine University Düsseldorf Universitätsstraße 1 40225 Düsseldorf Germany shirin.faraji@hhu.de; b Department of Chemistry, Universidad Autónoma de Madrid, Calle Francisco Tomás y Valiente, 7 28049 Madrid Spain juan.nogueira@uam.es; c Unit of Psychiatry, Department of Molecular Medicine, University of Siena Siena 53100 Italy; d IADCHEM, Institute for Advanced Research in Chemistry, Universidad Autónoma de Madrid, Calle Francisco Tomás y Valiente, 7 28049 Madrid Spain; e International Foundation Big Data and Artificial Intelligence for Human Development Via Galliera 32 40121 Bologna Italy

## Abstract

Psychedelic compounds can induce rapid-acting and long-lasting antidepressant benefits. Understanding the role of their hallucinatory effects is crucial for shaping the future trajectory of antidepressant drug development. Photoswitchable compounds targeting the 5-HT_2A_R offer precise spatio-temporal control over the activation of different downstream pathways. In this work, we computationally discovered PQ-azo-*N*,*N*-DMT (34), a photoswitch with improved features compared to the previously synthesized azo-*N*,*N*-DMT (1). The new compound shows tight binding to the 5-HT_2A_R, retaining all important interactions of lysergic acid diethylamide (LSD), exhibits positive membrane permeability, and has a strong red-shifted absorption that would allow photocontrol in the visible spectrum.

## Abstract 2

Molecular dynamics, umbrella sampling, and QM/MM were used for modelling binding, permeation, and photophysics of photoswitchable psychedelics, identifying features that tune absorption spectra and enhance receptor and membrane interactions.

## Graphical abstract

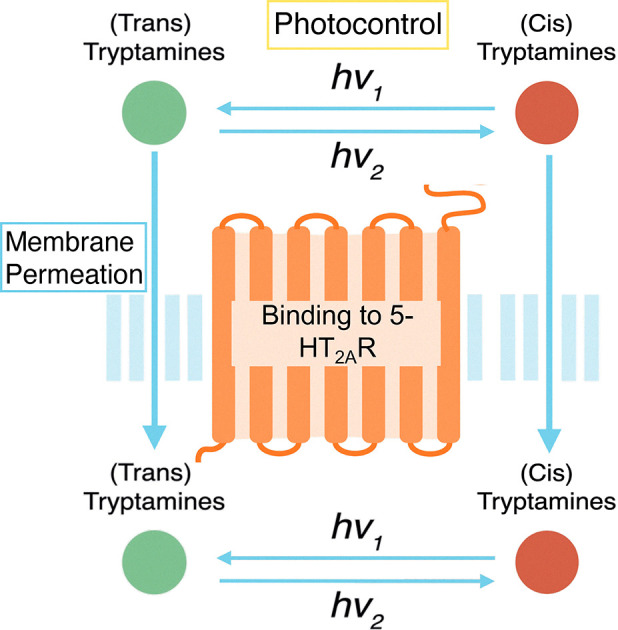

## 1 Introduction

**Fig. 1:**
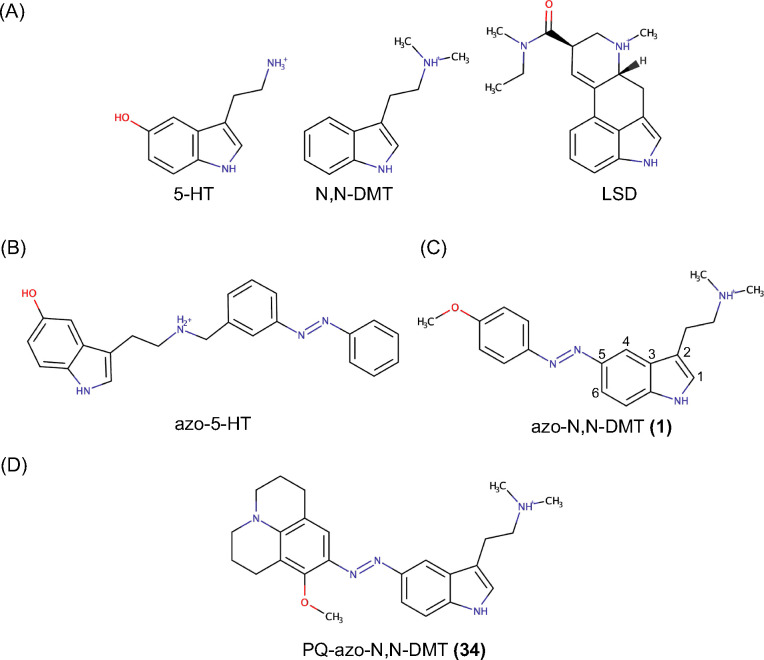
(A) Molecular structure of 5-hydroxytryptamine (5-HT), *N*,*N*-dimethyltryptamine (*N*,*N*-DMT) and lysergic acid diethylamide (LSD) (B) first class of photoswitchable 5-HT_2A_R (azo-5-HT). (C) Second class of photoswitchable 5-HT_2A_R (azo-*N*,*N*-DMT). (D) Newly discovered compound julolidine-azo-*N*,*N*-DMT (PQ-azo-*N*,*N*-DMT).

After decades of global constraint on research, the current “renaissance” of scientific interest in psychedelic compounds is one of the most promising areas in mental health.^1^ There is a significant growing body of preclinical^2–4^ and clinical evidence^5–9^ that psychedelic compounds can induce significant rapid-acting and long-lasting therapeutic effects, as opposed to typical psychiatric medications that require repeated exposure and have a longer onset of action.^10,11^ While serotonergic psychedelics, such as lysergic acid diethylamide (LSD) or *N*,*N*-dimethyltryptamine (*N*,*N*-DMT) (Fig. 1A) display intricate polypharmacology,^12^ they exhibit high affinity for the serotonin or 5-hydroxytryptamine (5-HT) receptors.^13^ The leading hypothesis of their psychoactivity and structural plasticity is through the activation of the 5-HT_2A_ receptor (5-HT_2A_R),^14–17^ despite recent findings suggesting that the antidepressant activity of 5-HT_2A_R agonists may be independent of their psychoactive properties^18–20^ or even independent of 5-HT_2A_R activation.^21^ It is of great interest for designing new improved antidepressants to understand whether the acute drug-induced experiences and antidepressant effects can be entirely separated, and demonstrate whether these subjective experiences are mere byproducts of psychedelic compounds or integral to their therapeutic efficacy.^22^

**Fig. 2:**
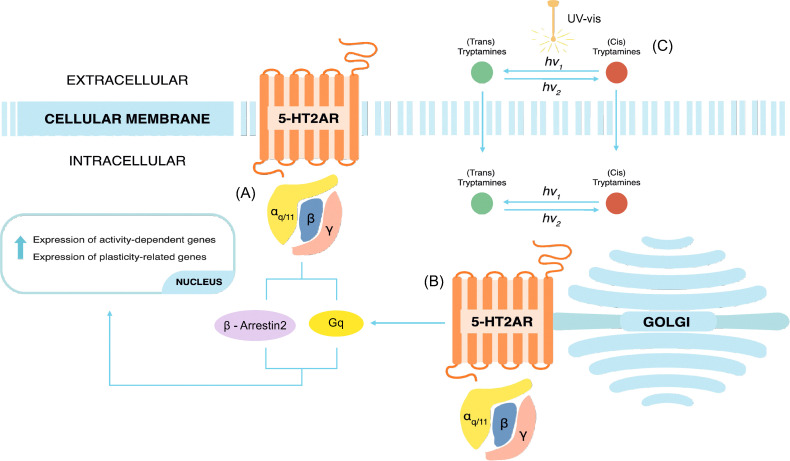
Schematic representation of the neurophysiological effects of classic psychedelics interacting with the 5-HT_2A_R. (A) Activation of the G_q_ and β-arrestin2 downstream pathways from 5-HT_2A_Rs located on the cellular membrane surface. (B) Activation of 5-HT_2A_Rs located on the Golgi membrane surface intracellularly. (C) Reversible activation of photoswitchable tryptamines that can activate both extra- and intracellular pools of 5-HT_2A_R.

In G-coupled protein receptors (GCPRs), similar compounds can stabilize different receptor conformations and induce downstream pathways with varying efficacy. This phenomenon is known as biased agonism or functional selectivity.^23,24^ Psychedelic compounds activate both G_q_ and β-arrestin2 pathways, which are the two main intracellular signaling pathways triggered by the binding of these compounds to the 5-HT_2A_R (Fig. 2A).^25^ Recent studies show that a certain G_q_-efficacy threshold is required to induce hallucinogenic effects in mice, while β-arrestin2 recruitment alone does not appear to be sufficient to induce these effects.^26^ In addition to a functional selectivity based solely on the ligand–receptor interaction, there is now considerable evidence that the location of the signal transduction can also influence functional selectivity, a phenomenon known as location bias.^27^ Indeed, the neuroplastic effects induced by classic psychedelics are primarily due to the activation of 5-HT_2A_R pools located intracellularly on the membrane of the Golgi apparatus, rather than on the cellular membrane surface^16^ (Fig. 2B).

In parallel with the development of biased agonists of the 5-HT_2A_R, photo-pharmacological compounds targeting GPCRs are being developed to achieve precise spatio-temporal control over the activation of different downstream pathways.^28^ Given the dynamic temporal nature of GPCR signaling,^29^ and that of the 5-HT_2A_R, it would be of great importance to achieve reversible photocontrol on the activation of G_q_ and β-arrestin2 pathways using a small drug^30^ (Fig. 2C). Recently, the first photoswitchable analog of 5-HT was developed, and the efficacy to different 5-HT subtypes receptors was tested *in vitro*.^31^ In this first generation of 5-HT photoswitches, the photosensitive azobenzene (AZ) domain was conjugated to the ethylamine side of 5-HT (Fig. 1B). The degree of G_q_ activation was evaluated with a fluorescent sensor to test, showing that a response was achieved with nM concentrations. Moreover, the compound showed preferential agonism in the *cis* configuration over the *trans* configuration on the 5-HT_2A_R, with no clear difference observed on the 5-HT_2B_R and 5-HT_2C_R. Despite being a great starting point, further optimization of the structure is needed to achieve greater resemblance with the endogenous compounds. To this end, a few months later, a second generation of 5-HTR photoswitches was developed by incorporating the indole core of *N*,*N*-DMT into the photosensitive domain (Fig. 1C). This design allowed one AZ ring to be part of the indole group, requiring only one additional aromatic ring to achieve light sensitivity.^32^ Based on prior knowledge of the photophysical properties of phenylazoindoles, which showed an undesirably fast thermal isomerization of the *cis* isomer when the phenyl ring was attached to the C_2_ or C_3_ carbons,^33^ the AZ addition was performed on the C_4_, C_5_, and C_6_ positions. To assess their ability to activate the 5-HT_2A_R *in vitro*, a β-arrestin2 recruitment assay was employed as a quantifiable measure of receptor activation. Addition of AZ at the C_5_ position and *para*-methoxy substitution, azo-*N*,*N*-DMT (1), demonstrated the greatest potency and reversible photocontrol over the receptor. The *cis* isomer enabled the activation of the receptor with sub-μM concentration and exhibited similar efficacy to *N*,*N*-DMT, while the *trans* isomer reduced receptor activation but was still found to bind at similar μmolar concentrations. Although the *trans* isomer can bind to the receptor at concentrations similar to the *cis* isomer, its ability to activate the receptor (efficacy) is significantly lower, indicating that the photosensitive molecule functions as an “efficacy switch”, maintaining selectivity for the 5-HT_2A_R without losing its specificity. An issue encountered was a slight residual activity in the *trans*-enriched state, attributed to incomplete (back)switching. To corroborate this hypothesis, it is essential to conduct an in-depth study of the photophysics of (1) and design compounds that exhibit a significant energy gap between the excitations responsible for the photoisomerization pathways. These compounds must also meet all the requirements for being effective agonists of the 5-HT_2A_R and permeate spontaneously though the cell membrane.

In this computational work, we characterized the binding mode, membrane permeability and photophysics of the recently synthesized (1), using molecular dynamics (MD), umbrella sampling (US), quantum mechanics/molecular mechanics (QM/MM) and free energy calculations. Our goal was to develop a compound that tightly binds to the orthosteric binding pocket (OBP) of the 5-HT_2A_R in at least one of the two isomers, with positive membrane permeability, a red-shifted wavelength of absorption, and a substantial energy gap between the two reversible photoisomerizations. From a pool of 160 compounds (80-*trans*, 80-*cis*), PQ-azo-*N*,*N*-DMT (34) (Fig. 1D) was found to have all the aforementioned characteristics and, thus, presents improved physico-chemical properties compared to (1).

## 2 Computational details

### 2.1 QM excited-state calculations

The construction of all 160 structures was performed using the IQmol Molecular Viewer.^34^ The selection of these compounds was guided by the study of Aleotti *et al.*, which evaluated similar systems with enhanced push and pull substituent strengths.^35^ Subsequently, a conformational search was carried out using a combination of the conformer–rotamer ensemble sampling tool (CREST) and command line energy sorting ensemble (CENSO).^36,37^ After an iterative metadynamics run performed by CREST, the 20 lowest energy conformers were input into CENSO. An initial pre-screening of the input conformers was performed through single-point energy calculations at the quantum mechanical level, using the B97-D3/def-SV(P) level of theory with the GFN2-xTB solvation model and a threshold energy of 4.0 kcal mol^−1^.^38,39^ Subsequently, more accurate electronic and solvation energies were calculated in a second screening at the r2SCAN-3c/def2-mTZVPP level of theory, with a threshold energy of 3.0 kcal mol^−1^.^37^ The geometries of the relevant conformers were initially optimized at the r2SCAN-3c/def2-mTZVPP level to obtain relatively accurate geometries rapidly for the entire molecular set coming from the previous screening. Then, only the lowest energy structure was reoptimized at the B3LYP/cc-pVDZ level of theory with D3 empirical dispersion and the polarizable continuum model, using a dielectric constant of 78.355 to simulate a water environment.^40^ Although the protocol could have ended at the r2SCAN-3c stage, we decided to use an hybrid functional to improve the geometry and also to ensure consistency with the level of theory subsequently used for the TD-DFT excited-state calculations. In addition, while standard hybrid functionals such as B3LYP may be inadequate for true long-range charge-transfer excitations, our study focused exclusively on tracking the relative red-shift of the local ππ* band, for which B3LYP-D3 remains a consistent choice. Fig. S1–S5 show the *trans* conformations of all the optimized compounds, while Tables S1–S5 list the energies and oscillator strengths of the first 10 singlet excited states (see SI).

### 2.2 Initial structures

The holo-structure of the 5-HT2AR bound to the non-selective agonist LSD was obtained from the RCSB-PDB (PDB ID: 6WGT).^25^ While alternative structures have been recently crystallized,^20^ the LSD-bound structure was deemed most suitable for the study due to the size of the ligands under investigation and the ergotamine domain which contains the structure of the tryptamine domain of the photoswitchable compounds. For both (1) and (34) the predominant titration state in an aqueous solution at neutral pH is the protonated form, since the p*K*_a_ is >7.8. Thus, it was selected for all simulations. The final geometries were used to compute restricted electrostatic potential (RESP) charges^41^ from the electrostatic potential at the Hartree Fock (HF)/6-31G* level, and these charges were embedded into MOL2 files generated with the AmberTools Antechamber suite.^42^ We adopted HF/6-31G* specifically to remain consistent with the FF19SB force-field parametrisation for aminoacids, which relies on RESP charges to reproduce electrostatic interactions in biomolecular simulations.^43^ Force constants for the dihedral angles (–C–N=N–C– and –C–C–N=N–) of the photoswitches were adopted from previous work.^44^ By aligning the indole group to the crystallized structure of LSD and considering insights from mutagenesis studies,^45–49^ the photoswitches were manually placed focusing on key features: the protonated tertiary amine nitrogen interacting with D155, the indole –N1 atom forming a hydrogen bond with S242, and the aromatic indole group engaging in hydrophobic interactions with W336, F339, and F340. Missing heavy atoms and hydrogens were added with the tleap module of the Amber20 package for the 5-HT_2A_R.^42^ All membrane–protein–ligand systems were constructed using CHARMM-GUI.^50^ The protein was oriented along the *z*-axis using the orientations of proteins in membranes database and the positioning proteins in membrane web server.^51^ Subsequently, N- and C-end groups were amidated and acetylated. The resulting structure was then embedded within a 1-palmitoyl-2-oleoyl-*sn-glycero*-3-phosphocholine (POPC) lipid bilayer with dimensions of 50 × 50 lipid components in the *xy* plane and solvated in a rectangular box with aqueous solvent and NaCl at a concentration of 0.15 mol L^−1^. Potential parameters for the protein, lipids, water, and ligands were taken from the FF19SB, Lipid17, TIP3P, and GAFF2, respectively.^43,52–54^

### 2.3 Molecular dynamics simulations

MD simulations for the solvated membrane–protein–ligand system were performed with the CUDA version of the AMBER20 package.^42^ First, energy minimization was carried out with the steepest descent method for 5000 steps followed by the conjugate gradient method for additional 5000 steps. Positional restraints were applied to the membrane–protein–ligand system gradually starting from 10, 7.5, 5, 2.5, 1 to 0.5 kcal (mol^−1^ Å^−2^) while heated from 0 to 303.15 K in six steps of 1250 ps, with the Langevin thermostat for a total of 7500 ps. The desired density was reached by running an equilibration in the NPT ensemble with a Monte Carlo barostat and a semi-isotropic pressure scaling for 4 ns for 500 ps. An unconstrained production run was carried out at 303.15 K for 5 independent copies of 1 μs for LSD, *cis*-(1), *trans*-(1). *Cis*-(1) and *trans*-(1) for a total simulation time of 25 μs. During the full MD protocol, a timestep of 2 fs was employed, and the cutoff radius and switching distance to compute the non-bonded interactions were set to 12.0 Å and 10 Å, respectively. Bond lengths involving hydrogen atoms were kept fixed using the SHAKE algorithm.^55,56^ Electrostatic interactions were calculated using the Ewald particle mesh method with a grid spacing of 1 Å.^57^

### 2.4 End state free energy calculations

The relative binding free energy of all the systems was calculated with the 1 average molecular mechanics generalized Born surface area (MMGBSA) method.^58^ Bond radii were set to mbondi2 based on igb = 5 of the AMBER implementation,^59^ and salt concentration was set to 0.15 mol L^−1^. A total of 500 equidistant snapshots were selected to calculate the MMGBSA total binding free energy for each molecule. In addition, a pairwise residue free-energy decomposition analysis was performed to obtain the contribution of each residue–ligand interaction to the total binding free energy. The decomposition was performed by considering residues within a radius of 7 Å distance from the ligand.

### 2.5 Membrane permeation

The photoswitches *cis*-(1), *trans*-(1), *cis*-(34), and *trans*-(34) were then embedded within a POPC lipid bilayer with dimensions of 50 × 50 lipid components in the *xy* plane and solvated in a rectangular box with aqueous solvent and NaCl at a concentration of 0.15 mol L^−1^. Potential parameters for the lipids, water, and ligands were taken from the Lipid17, TIP3P, and GAFF2, respectively.^52–54^ An energy minimization was carried out with the steepest descent method for 5000 steps followed by the conjugate gradient method for additional 5000 steps. The membrane and the ligand were subjected to positional restraints of 10 kcal (mol^−1^ Å^−2^) while being heated in the NVT ensemble from 0 to 303.15 K with the Langevin thermostat with a relaxation time of 1 ps for a total simulation time of 750 ps and 2 fs timestep. Then, an NPT simulation was evolved with a Monte Carlo barostat and semi-isotropic pressure scaling and a Langevin thermostat with a relaxation time of 1 ps for 500 ps with a timestep of 2 fs. Subsequently, a single harmonic restraint of 2.5 kcal (mol^−1^ Å^−2^) was applied to maintain the distance between the center of mass (CoM) of the POPC bilayer and the initial position of the ligand at *z* = 0 Å during a production run of 100 ns. A pulling run was conducted to gradually diffuse the ligand from the membrane center (*z* = 0 Å) to the bulk water phase (*z* = 36 Å). The pulling rate employed was 1 Å ns^−1^. This pulling process was performed to obtain initial geometries for subsequent US simulations, which are described later. Pulling the ligand from the center of the membrane towards the water phase is beneficial for achieving faster convergence of the PMF compared to pulling from the water phase into the membrane.^60^ During the full MD protocol, a timestep of 2 fs was employed, the cutoff radius and switching distance to compute the non-bonded interactions were set to 12 Å and 10 Å, respectively. Bond lengths involving hydrogen atoms were kept fixed using the SHAKE algorithm.^55,56^ Electrostatic interactions were computed using the particle-mesh Ewald method with a grid spacing of 1 Å.^57^ US was employed to explore the diffusion pathway from the center of the bilayer to the bulk solvent.^61^ In this approach, a single harmonic restraint with a force constant of 2.5 kcal (mol^−1^ Å^−2^) was applied to restraint the value of the reaction coordinate (RC), which was defined as the *z*-axis component of the distance between the CoM of the POPC bilayer and the CoM of the compounds. The RC was divided into 45 windows spaced at intervals of 0.8 Å along the *z*-direction from the center of the membrane (*z* = 0 Å) to the water phase (*z* = 36 Å). In each window, a MD trajectory was evolved for 50 ns, and the analyses were performed on the full 50 ns of each simulation. Energy convergence analyses were based on previous findings of permeation of tryptamines in a POPC lipid bilayer.^62^ The weighted histogram analysis method^63,64^ was used to calculate the potential of mean force (PMF) along the RC for each compound based on the data obtained from each US simulation, which allowed to determine the logarithm of the effective permeability (log *P*_eff_) as illustrated in previous works.^65,66^

### 2.6 QMMM excited states calculations

The absorption spectra of *cis*-(1), *trans*-(1), *cis*-(34) were computed in the 5-HT_2A_R using QM/MM calculations performed on 100 snapshots taken equidistantly from the 500 frames used for MMGBSA. The MoBioTools package^67^ was used to extract the geometries, split the system into the QM and MM regions, and prepare the input for the QM/MM calculations using the electrostatic embedding scheme. The QM region consisted of the photoswitches and is modeled using TD-DFT including the first 10 singlet excited states at B3LYP-D3/cc-pvdz level of theory, while the MM region consisted of explicit point charges that included the rest of the system.^68,69^

## 3 Results and discussion

### 3.1 QM excited-state calculations

A fundamental characteristic required for a photoswitch to be effectively used in a biological environment for receptor activation and deactivation is the ability to undergo photoisomerization at two distinctly separated wavelengths.^70^ Furthermore, during the optimization of the chemical structure, it is essential to aim for a pronounced red shift in the absorption spectrum, enhancing its potential for *in vivo* applications.^71^ Starting with (1), in this work, a systematic *para* substitution was conducted to create push–pull AZ compounds, promising candidates for achieving red-shifted absorption.^70^ In the *trans* isomer, the simultaneous destabilization of the highest occupied π orbital (electron-donating substituents) and stabilization of the lowest unoccupied π* orbital (electron-withdrawing substituents) leads to a red shift of the bright S_2_ (π → π*) absorption, reducing the energy gap with S_1_ (n → π*), known to be the most productive pathway for photoisomerization.^35^ The *cis* state instead demonstrates a higher quantum yield (QY) of photoisomerization following the excitation to the S_1_ state compared to the *trans* isomer, associated with a barrierless and steeper minimum energy path along the torsional motion,^72^ a characteristic observed not only in AZ but also maintained in push–pull systems. Based on this, we hypothesize that our compounds bound to the 5-HT_2A_R would exhibit a similar behavior, with the *cis* isomer demonstrating a higher QY of photoisomerization due to a steeper and barrierless minimum energy path along the torsional coordinate. Additionally, *ortho*-methoxy substituents were introduced in all possible combinations (Fig. S1–S5), resulting in a red shift primarily in the S_1_ (n → π*) state.^73^ This modification also slows down the thermal relaxation process, which is known to occur extremely fast in push–pull azobenzenes.^74^

Considering only *para* substitutions, the BZ-azo-*N*,*N*-DMT class of compounds (Table S2) (see SI) was particularly notable among various groups. These compounds exhibited a significant red-shifted absorption of approximately 1.01 eV for the S_2_ (π → π*) state in the *trans* isomer. As a consequence, this state becomes lower in energy than the S_1_ (n → π*) state (not the S_1_ anymore), leading to substantial orbital mixing between the two states, while a red shift of 0.32 eV was observed for the S_1_ (π/n → π*) state of the *cis* isomer, defined as π/n because of a substantial orbital mixing. Additionally, the PQ-azo-*N*,*N*-DMT class (Table S3) (see SI) exhibited a significant red shift of 0.61 eV in the S_2_ (π → π*) transition, while the other classes showed behavior similar to (1).

**Fig. 3:**
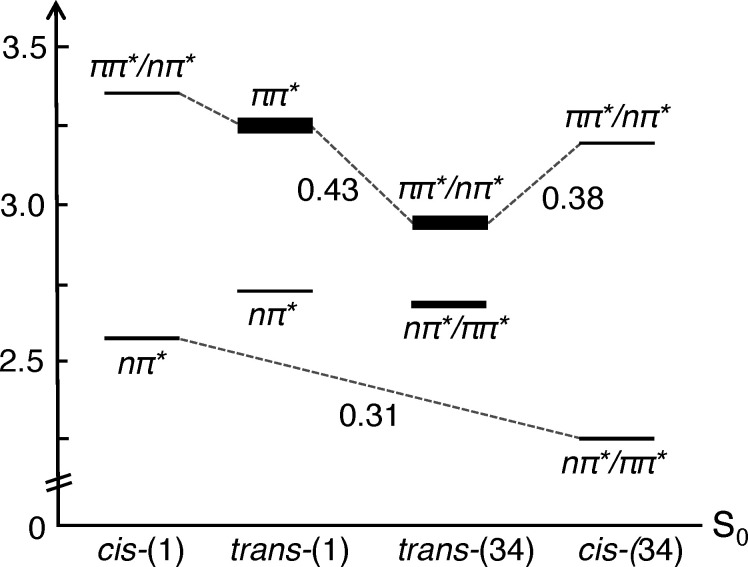
Energies and oscillator strengths (represented by the width of the lines in a qualitative way) of the relevant electronic excited states of the species azo-*N*,*N*-DMT (1) and PQ-azo-*N*,*N*-DMT (34) in their *cis* and *trans* configurations.

Intrigued by the behavior of BZ-azo-*N*,*N*-DMT and PQ-azo-*N*,*N*-DMT, we investigated whether these two classes demonstrated a clear separation between absorption and potential photoisomerization in both the *trans*- and *cis*- isomers. Although all BZ compounds exhibited the strongest red-shifted absorption, the bright states of the *trans* and *cis* isomers almost coincide at the same energy, potentially leading to a mixed photostationary distribution and residual activity of the 5-HT_2A_R. However, for PQ-azo-*N*,*N*-DMT (Table S3), the *ortho*-substituted compound (34) presented a promising scenario. Its *trans* isomer exhibited a significant red shift, from 3.24 eV to 2.81 eV compared to (1), resulting in a pronounced alteration in the character of the S_2_ (π → π*) state (Fig. 3). This shift caused a blending with the closely located S_1_ (n → π*) state at 2.70 eV. In contrast, the *cis* isomer displayed its first excited state, S_1_ (π/n → π*), at 2.26 eV, introducing a distinct red shift compared to *cis*-(1), which exhibits this state at 2.57 eV (Fig. 3). Given those changes, a larger energy gap of 0.38 eV between the *trans*-(34) bright state and the *cis*-(34) S_2_ (π/n → π*) state, which might prevent the mixing of the two isomers when the molecule is excited. In comparison, for the (1) species, the energy difference between the bright states of the two isomers is only 0.14 eV. The scenario of (34) creates an opportunity for photoisomerization from *trans* to *cis* at approximately 2.70 eV and from *cis* to *trans* at around 2.25 eV, minimizing mixing between the two isomers. Since the (34) species presents appropriate absorption features, its adequacy as HT_2A_R inhibitor will be evaluated in more detail and compared with (1) in the following sections.

### 3.2 Binding to the 5-HT_2A_R

To validate whether the selected compounds could bind and maintain the canonical binding pose of tryptamines and ergotamines within the OBP of the 5-HT_2A_R, the *N*,*N*-DMT domain of (1) and (34) in both isomers was superimposed onto the rigid tryptamine domain of LSD using VMD.^75^ Both LSD and the photoswitches remained tightly bound to the OBP for the whole simulation time of 5 μs, as indicated by the root mean square deviations (Fig. S6) (see SI). The MMGBSA method was employed to estimate the relative binding free energies (Δ*G*_tot_) and further decompose it into van der Waals (Δ*G*_vdW_) and electrostatic (Δ*G*_el_) interactions between the ligand and receptor, as well as changes in the solvation energy (Δ*G*_sol_) of the receptor and ligand complex by the implicit solvent upon binding. Additionally, each energy term was further decomposed into pairwise residue–ligand binding free energies to identify the main amino acids contributing to the binding process. Both *cis*-(34) and *trans*-(34) exhibit more favorable Δ*G*_vdW_ than (1) and LSD due to their bulkier domains that establish numerous hydrophobic residues in the pore (Table 1). This stabilization results in more favorable Δ*G*_tot_ values compared to LSD and both *trans*- and *cis*-(1). In addition, for both (1) and (34), the *trans* isomer exhibits less favorable Δ*G*_el_ compared to the *cis*, indicating an overall different character of interaction inside the OBP.

**Table 1:** Binding free energy decomposition (Δ*G*_tot_) into van der Waals (Δ*G*_vdW_), electrostatic (Δ*G*_el_), and solvation (Δ*G*_sol_) components for all compounds under investigation. Logarithm of the effective permeability (log *P*_eff_) of all the compounds permeating into a POPC lipid bilayer

Compound	Δ*G*_vdW_	Δ*G*_el_	Δ*G*_sol_	Δ*G*_tot_	Log *P*_eff_
LSD	−47.8	−73.7	66.8	−54.8	
*Cis*-(1)	−49.7	−78.2	71.5	−56.4	1.36
*Trans*-(1)	−50.6	−69.9	66.1	−54.4	1.49
*Cis*-(34)	−60.4	−73.0	74.5	−65.9	1.34
*Trans*-(34)	−65.6	−68.4	67.2	−66.9	1.51

**Fig. 4:**
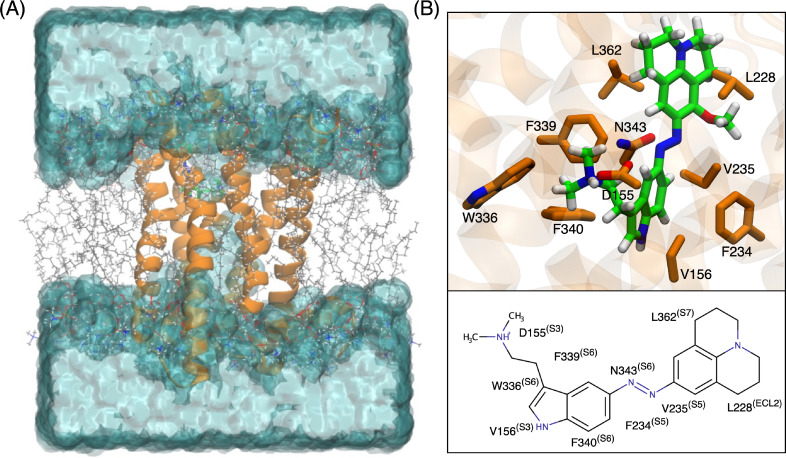
(A) Representation of PQ-azo-*N*,*N*-DMT (34) (green) inserted into the orthosteric binding pocket (OBP) of the 5-HT_2A_R embedded in a 1-palmitoyl-2-oleoyl-*sn-glycero*-3-phosphocholine (POPC) lipid bilayer (gray) and water (cyan). (B) The 10 residues and the corresponding helices (S3, S5, S6, S7) along with extracellular loop 2 (ECL2) that contribute to the binding of PQ-azo-*N*,*N*-DMT (34) in the OBP of the 5-HT_2A_R.

**Fig. 5:**
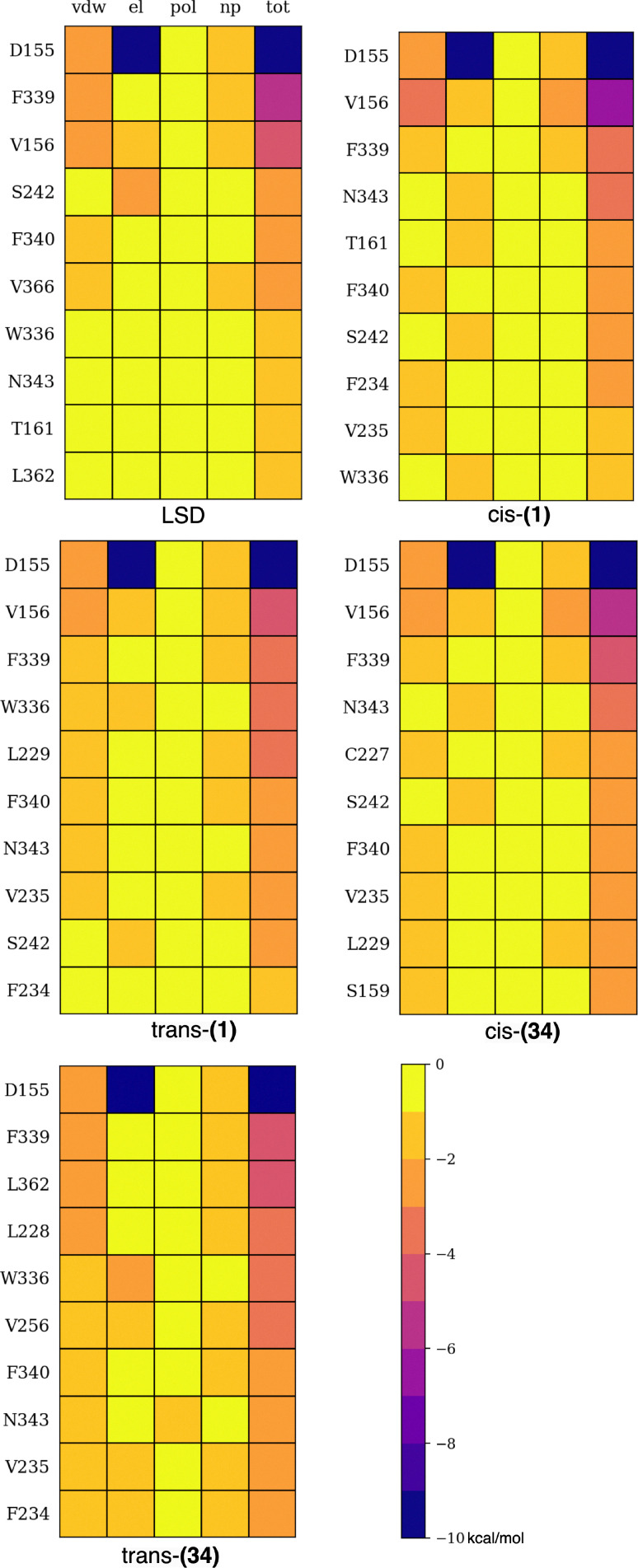
Pairwise residue decomposition of the van der Waals (Δ*G*_vdW_), electrostatic (Δ*G*_el_), polar (Δ*G*_pol_), non-polar (Δ*G*_np_) solvation and total binding free energy (Δ*G*_tot_) including the major 10 residue–ligand interactions for all the compounds.

The OBP is positioned approximately 10 Å inside the 5-HT_2A_R protein from the extracellular medium. Helices S3, S5, S6, S7, and extracellular loop 2 (ECL2) fold toward the center of the pore, constituting the most implicated protein fragments in drug binding. The S3 helix accommodates the ethylamine domain of all the compounds, where D155 plays a crucial role in anchoring it to the pocket through a salt bridge between the ^+^NH(CH_3_) group of the ligands and the negatively charged COO^−^ side chain of D155 (Fig. 4B).^76^ This interaction is confirmed by the strong electrostatic interactions observed in the energy decomposition analysis (Fig. 5). Continuing along the S3 helix, V156 creates alkyl–π interactions with the aromatic indole (Fig. 4B). Despite not being part of mutagenesis studies, computational studies have previously identified V156 as a robust contributor to the binding of 25X-NBOMEs and LSD.^77^ S159 forms interactions with both LSD and the photoswitches, where the –OH side chain creates a hydrogen bond with the tertiary alkyl amines of the compounds. This interaction is observed within the first 10 residues only in *cis*-(34) (Fig. 5). In contrast, primary amines such as 5-HT are known to interact more strongly with this residue, forming multiple hydrogen bonds.^25,47^ In the S5 helix, S242, a unique residue of the 5-HT_2A_R among the 5-HTRs family, engages in hydrogen bonding with the indole –N1 of all compounds through its –OH side chain, except for *trans*-(34), which is slightly displaced (Fig. 5).^78^ The photoswitches experience additional stabilization through interactions with L228 and L229 in ECL2, which appears to be weaker in LSD and not shown within the first 10 energy decomposed residues (Fig. 5). L229, identified as a “lid” of the ECL2, was shown to induce receptor closure and enhance the stabilization of LSD.^25,79^ Additionally, F234 and V235 at the extracellular edge of the S5 helix contribute to this stabilization.

The hydrophobic S6 region participating in the OBP enhances ligand interaction through cation–π and π–π interactions with the indole group of all the ligands. Four key aromatic residues, namely W336, F339, F340, and N343 contribute significantly to the binding of all compounds. W336, referred to as the “toggle switch,” has recently been shown to guide G_q_ efficacy through an alteration of the *χ*_2_ dihedral angle in the class of 25CN-NBx compounds.^26^ In our dynamics of tryptamines, the *χ*_2_ dihedral angle remained at its initial value of the 5-HT_2A_R-LSD holo-structure. F340 engages in a T-shaped ππ interaction with the indole aromatic group, where the van der Waals term dominates. Mutagenesis studies have indicated that the F340L mutation significantly reduces the affinity of various 5-HT_2A_R agonists, including 5-HT and 5MeO-*N*,*N*-DMT. However, the F340Y mutation, maintaining an aromatic residue at the position, behaves similarly to the native receptor, emphasizing the requirement for an aromatic residue rather than specifically phenylalanine.^46,80^ Finally, *trans*-(34) exhibits strong interactions with L362 in the S7 helix, a residue that only weakly interacts with LSD. Thus, overall, the four photoswitches maintain the key interactions within the most relevant helices and exhibit comparable affinity to LSD in the 5-HT_2A_R. It is important to note that (34), identified in the previous section as the compound with the most appropriate absorption features, displays the tightest binding to the receptor.

### 3.3 Membrane permeation

**Fig. 6:**
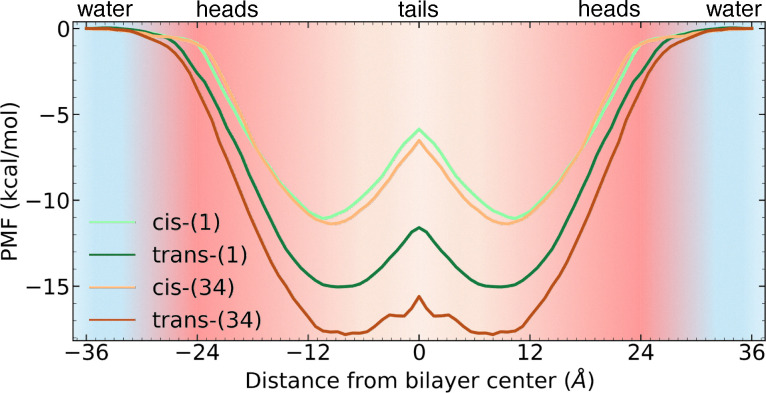
Symmetric PMF for the photoswitches permeating into a POPC lipid bilyer. The water phase (cyan) extends from ±36 Å to ±25 Å, the head groups (red) from ±24 Å to ±13 Å, and the lipid tails from ±12 Å to 0 Å.

Once we have observed in the MD simulations and free energy calculations that the photoswitches were able to retain the key interactions in the OBP, we simulated their permeation within a POPC lipid membrane, which is a necessary step to reach the intracellular 5-HT_2A_R. Table 1 presents the effective permeability, log *P*_eff_ values obtained from the PMF. A positive log *P*_eff_ indicates that the compound is permeable, while a negative value indicates impermeability. All four compounds exhibit positive permeability, with the *trans* isomers having slightly higher values. All the compounds permeate from the bulk water (*z* = 36–26 Å) phase to the membrane's head groups (*z* = 25–12 Å) in a barrierless manner (Fig. 6), until reaching a minimum at 8–11 Å away from the membrane's center, sitting just below the head groups, in a region of medium polarity. Indeed, the *cis* isomers, in particular *cis*-(1), exhibit the closest minimum to the head groups, indicating an overall higher polarity, which can be qualitatively associated with the higher Δ*G*_el_ inside the OBP. The *trans* isomers display a much deeper minimum and a smaller energy barrier at the membrane's center (*z* = 0 Å), associated with a greater log *P*_eff_ (around 1.50) in comparison to the *cis* isomers (around 1.35). Previously reported values of *N*,*N*-DMT in a POPC lipid bilayer display a very similar value (log *P*_eff_ = 1.59). Thus, the addition of an azobenzene domain and its *ortho*–*para* substituents do not significantly affect the overall permeability of the compound. This suggests that both (1) and (34) can easily permeate, activate intracellular pools of 5-HT_2A_Rs, and in principle induce neuroplastic effects.^16^

### 3.4 Absorption spectrum including sampling and biological environment

**Fig. 7:**
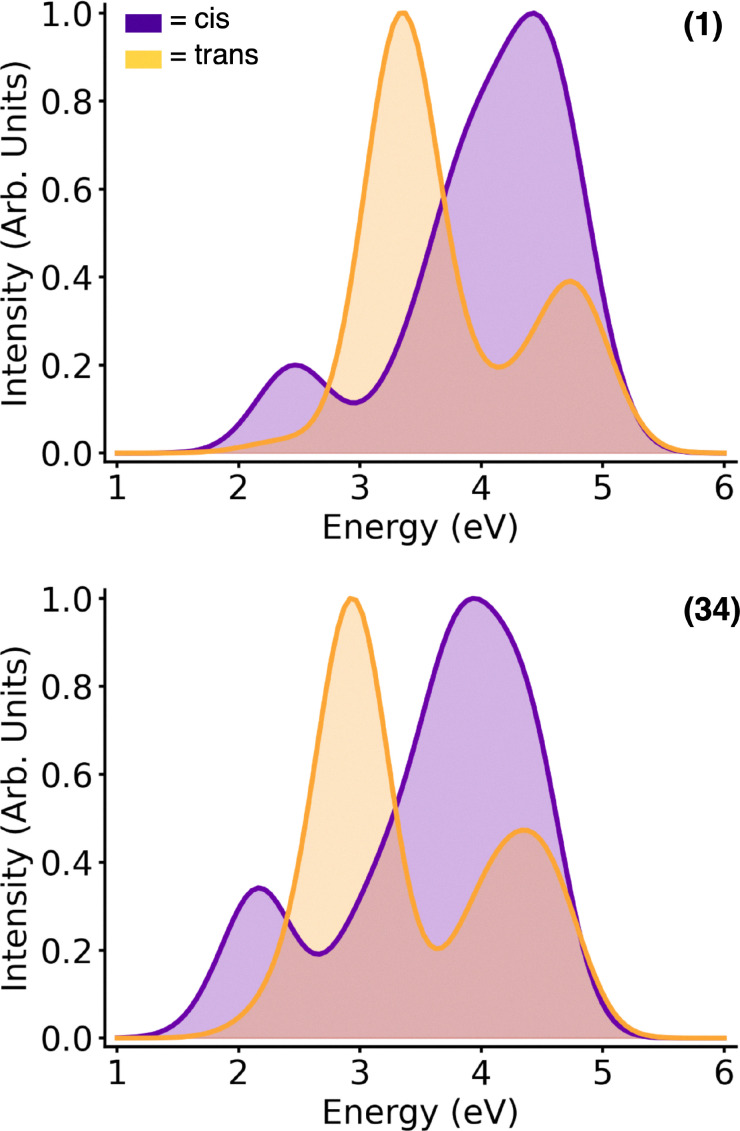
Absorption spectrum of the *trans*- and *cis*- azo-*N*,*N*-DMT (1) (top) and *trans*- and *cis*- PQ-azo-*N*,*N*-DMT (34) (bottom).

Inspired by the static TD-DFT calculations that showed a red-shifted absorption for (34), in comparison to (1), we computed the absorption spectra of all four isomers embedded in the OBP of the 5-HT_2A_R (Fig. 7). The static calculations were computed at the optimized geometry with implicit solvation, while the QM/MM calculations were computed from an ensemble of the MD snapshots taken from the production run, allowing the inclusion of the protein environment and vibrational sampling, which may strongly modify the electronic structure of chromophores.^81–85^ For (34), a significant red shift is observed for both *trans* and *cis* isomers with respect to (1), consistent with predictions from the QM static calculations. In *trans*-(34), the first absorption band shows a maximum at 2.95 eV, slightly blue-shifted in comparison to the QM calculations, while in *trans*-(1) the first absorption band maximum is found at 3.25 eV, in complete agreement with the QM calculations. In the *cis* isomer, (34) and (1) display the first absorption band maxima at 2.25 and 2.57 eV, in great agreement with QM calculations. The energy gap between *trans* and *cis* (34) absorption (Δ*E* = 0.70 eV) is similar to that between the *trans* and *cis* (1) (Δ*E* = 0.68 eV). Therefore, in both compounds, the independent excitation of *cis* and *trans* isomers is possible avoiding the mixture of both photoisomers. However, the two isomers of the newly designed compound (34) can be excited with light of less energy than (1), a desired feature of a photoswitch aimed to be employed in *in vivo* systems, as explained above.

## 4 Conclusion

Photo-pharmacological compounds targeting GPCRs enable precise spatio-temporal control over the activation of different downstream pathways. The reversibility of the photoisomerization process is a crucial property of AZ-based agonists, enabling precise, light-controlled modulation of biological systems. Within the context of 5-HT_2A_R, an ideal scenario would involve a molecule that activates a specific pathway, such as G_q_ or β-arrestin, in one isomer while deactivating it in the other. Alternatively, a compound capable of activating the G_q_ pathway in one isomer and the β-arrestin pathway in the other isomer would be highly advantageous. However, before reaching such an exquisite control, it is important to design photoswitches that absorb at low energies (large wavelengths) and can photoisomerize from *trans* to *cis* and from *cis* to *trans* using distinct wavelengths and, thus, avoiding the present of both isomers simultaneously.

In this work, we identified a compound, (34), using QM/continuum and QM/MM calculations, which exhibits a significant red-shifted absorption of around 0.40 eV in both isomers relative to (1) and a favorable energy gap between the *trans* and *cis* excitation, potentially minimizing residual activity. MD and free energy calculations highlight the tight binding of both (1) and (34) to the OBP, sharing essential residue interactions with the recently crystallized 5-HT_2A_R-LSD complex. The calculated PMF and log *P*_eff_ values closely resemble those of previously simulated *N*,*N*-DMT, with slightly greater permeation in favor of the *trans* isomers for both molecules, implying that both (1) and (34) have the potential to permeate the neuronal membrane and activate intracellular pools of 5-HT_2A_Rs.

This new photopharmacological compound can serve as a starting point for the selective activation of different signaling pathways for the 5-HT_2A_R. It is important to note that although we have calculated vertical excitations both in implicit solvent and within the protein complex, our current study does not include a detailed mechanistic analysis of the photoisomerization process. Investigating this mechanism will should a primary focus of future work, as it is essential for accurately determining the quantum yield of photoisomerization. Also, at present, light-activated drugs can be used only in tissues we can illuminate without surgery. A good example is KIO-301, now in phase II trials for retinitis pigmentosa: the retina is naturally accessible to light, so no implants are required.^86^ No approved device can deliver sufficient photon flux to deep structures without inserting optical fibres. A high-power functional near infrared spectroscopy array operating at 700–850 nm might switch red-shifted molecules in the outermost millimetres of cortex, but it cannot reach sub-cortical targets.^87^

## Conflicts of interest

There are no conflicts to declare.

## Supplementary Material

CP-027-D5CP01252J-s001

## Data Availability

Supplementary information: Images of the *trans* isomers of all the optimized compounds, and the energies and oscillator strengths of the first 10 singlet excited states for both *trans* and *cis* isomers of all the optimized compounds. Root Mean Squared Deviation of all the compounds inside of the orthosteric binding pocket and absorption spectrum of the *trans*- and *cis*- azo-*N*,*N*-DMT (1) (top) and *trans*- and *cis*- PQ-azo-*N*,*N*-DMT (34) (bottom) in nanometers. See DOI: https://doi.org/10.1039/d5cp01252j Data for this article, including input files for molecular dynamics simulations, ligand/protein binding free energy, and potential of mean force profiles for membrane permeation are available at the Zenodo repository at https://zenodo.org/records/15117909.
