# Supplementary material for: Computational design of an improved photoswitchable psychedelic based on light absorption, membrane permeation and protein binding
Source: Phys Chem Chem Phys. 2025 Aug 8;27(36):19454–64. doi: 10.1039/d5cp01252j (PMC12396349; doi:10.1039/d5cp01252j)
Supplement: CP-027-D5CP01252J-s001 [file CP-027-D5CP01252J-s001.pdf]

Supplementary Information (SI) for Physical Chemistry Chemical Physics.
This journal is © the Owner Societies 2025

Supporting Information

Computational Design of an Improved Photoswitchable Psychedelics Based on Light Absorption, Membrane Permeation and Protein Binding.

Vito F. Palmisano[a],[b], *Claudio Agnorelli*[c], *Shirin Faraji*[a], *Juan José Nogueira Pérez*[b]

[a] *Vito F. Palmisano, Shirin Faraji**
Institute of Theoretical and Computational Chemistry, Heinrich Heine University Düsseldorf, Universitätsstraße 1, 40225 Düsseldorf, Germany
E-mail: shirin.faraji@hhu.de

[b] V. F. Palmisano, J. J. Nogueira*
Department of Chemistry, Universidad Autonoma de Madrid, Madrid 28049, Spain
IADCHEM, Institute for Advanced Research in Chemistry, Universidad Autonoma de Madrid, Madrid 28049, Spain
E-mail: juan.nogueira@uam.es

[c] C. Agnorelli
Unit of Psychiatry, Department of Molecular Medicine, University of Siena, Siena 53100, Italy

Table of Contents

2. Chemical Structures and Singlet Excited States Calculations *3*

2.1 Azo-N,N-DMT 3

2.2 BZ-azo-N,N-DMT 5

2.3 PQ-azo-N,N-DMT 7

2.4 NH2-azo-N,N-DMT 9

2.5 NOCH3-azo-N,N-DMT 11

2. Chemical Structures and Singlet Excited States Calculations

2.1 Azo-N,N-DMT

(1)

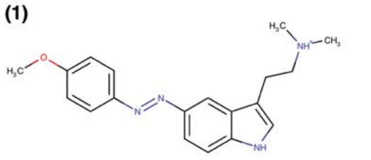

(2)

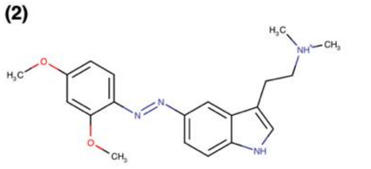

(3)

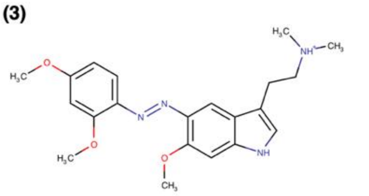

(4)

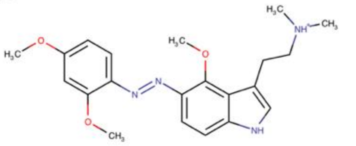

(5)

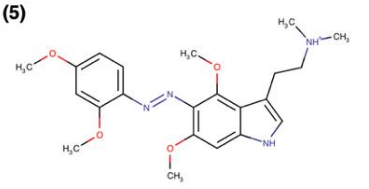

(6)

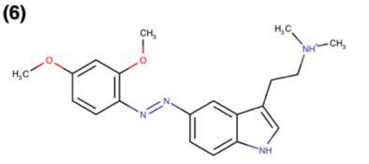

(7)

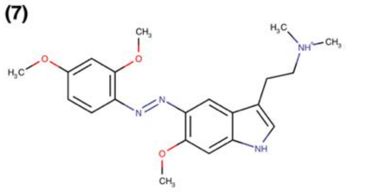

(8)

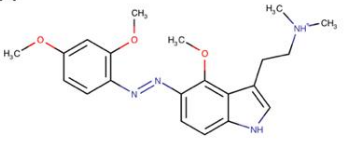

(9)

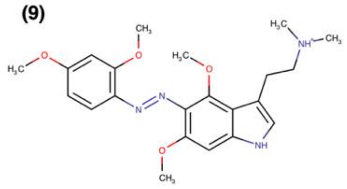

(10)

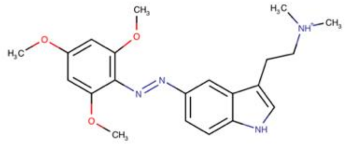

(11)

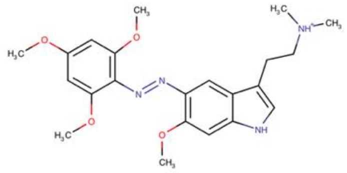

(12)

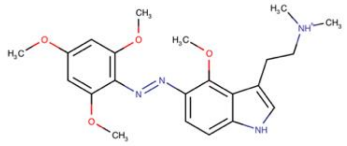

(13)

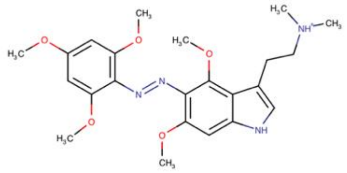

(14)

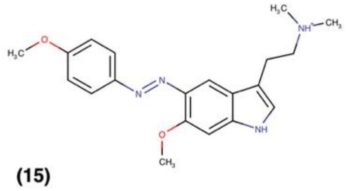

(15)

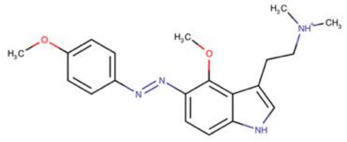

(16)

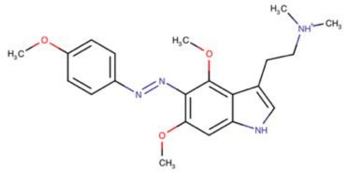

Figure S1: Chemical structure of the azo-N,N-DMT **(1)** to **(16)** in the trans isomer.

Table S1: Energies and oscillator strengths for the first 10 singlet excited states for the azo-N,N-DMT **(1)** to **(16)** compounds.

Trans																
	E(eV)	f	E(eV)	f	E(eV)	f	E(eV)	f	E(eV)	f	E(eV)	f	E(eV)	f	E(eV)	f
	1		2		3		4		5		6		7		8	
S_1	2.73	0.001	2.68	0.001	2.56	0.137	2.61	0.001	2.47	0.055	2.63	0	2.51	0.04	2.59	0
S_2	3.25	1.149	3.11	1.131	2.9	0.161	3.01	0.937	2.99	0.142	3.29	1.17	2.98	0.285	3.13	0.878
S_3	3.41	0.089	3.35	0.016	3.04	0.633	3.24	0.141	3.13	0.903	3.53	0.038	3.18	0.699	3.44	0.277
S_4	4.28	0.006	3.88	0.069	3.8	0.032	3.73	0.046	3.91	0.049	3.92	0.103	3.83	0.042	3.91	0.083
S_5	4.29	0.005	4.31	0.058	4.14	0.047	4.23	0.022	4.28	0.028	4.4	0.05	4.25	0.034	4.36	0.062
S_6	4.47	0.064	4.33	0.037	4.49	0.066	4.38	0.064	4.42	0.06	4.41	0.001	4.53	0.078	4.47	0.048
S_7	4.67	0	4.56	0	4.72	0.177	4.66	0.002	4.66	0.031	4.43	0.055	4.71	0.106	4.52	0.002
S_8	4.9	0.091	4.8	0.217	4.81	0.005	4.79	0.136	4.71	0.125	4.89	0.327	4.77	0.052	4.72	0.008
S_9	4.91	0.361	4.96	0.154	4.85	0.044	4.92	0.06	4.79	0.1	4.91	0.002	4.82	0.167	4.86	0.155
S_{10}	4.95	0.008	5.14	0.001	4.86	0.083	4.97	0.235	4.88	0.015	4.97	0.064	4.93	0.018	4.88	0.03
Cis																
	E(eV)	f	E(eV)	f	E(eV)	f	E(eV)	f	E(eV)	f	E(eV)	f	E(eV)	f	E(eV)	f
	1		2		3		4		5		6		7		8	
S_1	2.57	0.069	2.53	0.101	2.55	0.095	2.54	0.074	2.56	0.088	2.57	0.123	2.6	0.096	2.51	0.156
S_2	3.39	0.008	3.44	0.014	3.19	0.024	3.19	0.014	3.04	0.024	3.45	0.008	3.2	0.019	3.23	0.005
S_3	3.8	0.056	3.72	0.104	3.6	0.042	3.58	0.048	3.49	0.05	3.67	0.094	3.56	0.073	3.56	0.166
S_4	3.91	0.097	3.85	0.26	3.83	0.335	3.82	0.204	3.75	0.234	3.89	0.157	3.88	0.141	3.82	0.136
S_5	4.14	0.117	4	0.015	4.04	0.026	4.05	0.014	4.14	0.056	4.04	0.016	4	0.021	4.07	0.055
S_6	4.3	0.019	4.08	0.064	4.23	0.047	4.28	0.116	4.29	0.074	4.07	0.079	4.21	0.118	4.14	0.034
S_7	4.57	0.012	4.59	0.098	4.57	0.1	4.56	0.031	4.59	0.16	4.6	0.055	4.57	0.153	4.59	0.085
S_8	4.72	0.158	4.69	0.1	4.69	0.162	4.64	0.074	4.67	0.021	4.73	0.197	4.73	0.044	4.68	0.148
S_9	4.92	0.004	5.02	0.046	4.86	0.021	4.91	0.005	4.8	0.009	4.99	0.035	4.83	0.025	4.97	0.013
S_{10}	5.02	0.01	5.13	0.016	4.94	0.013	4.92	0.023	4.88	0.006	5.16	0.018	5.11	0.046	5.02	0.024

Trans																
	E(eV)	f	E(eV)	f	E(eV)	f	E(eV)	f	E(eV)	f	E(eV)	f	E(eV)	f	E(eV)	f
	9		10		11		12		13		14		15		16	
S_1	2.45	0.047	2.48	0.017	2.42	0.032	2.5	0.007	2.39	0.008	2.6	0.002	2.69	0.006	2.53	0.034
S_2	3.09	0.086	3.12	0.77	2.98	0.481	3.13	0.962	3.08	0.094	3.06	0.195	3.1	0.67	2.98	0.062
S_3	3.32	0.986	3.37	0.23	3.14	0.473	3.39	0.182	3.25	1.007	3.34	1.041	3.28	0.49	3.25	1.066
S_4	3.95	0.075	3.57	0.037	3.57	0.034	3.6	0.036	3.76	0.048	4.33	0.003	4.23	0.014	4.27	0.009
S_5	4.31	0.032	4.29	0.031	4.22	0.028	4.3	0.051	4.34	0.02	4.33	0.009	4.24	0.003	4.29	0.001
S_6	4.4	0.059	4.34	0.101	4.44	0.039	4.38	0.035	4.36	0.015	4.54	0.018	4.47	0.071	4.52	0.054
S_7	4.62	0.054	4.44	0.083	4.6	0.104	4.44	0.031	4.59	0.065	4.6	0.065	4.75	0.009	4.65	0.012
S_8	4.69	0.062	4.87	0.093	4.8	0.15	4.83	0.189	4.7	0.092	4.72	0.002	4.83	0.036	4.72	0.167
S_9	4.75	0.168	4.99	0.026	4.93	0.021	5.01	0.057	4.71	0.078	4.74	0.277	4.86	0.238	4.75	0.064
S_{10}	4.96	0.008	5.11	0.026	4.95	0.041	5.02	0.071	4.78	0.088	4.86	0.011	4.94	0.168	4.86	0.015
Cis																
E(eV)	f	E(eV)	f	E(eV)	f	E(eV)	f	E(eV)	f	E(eV)	f	E(eV)	f	E(eV)	f	E(eV)
	9		10		11		12		13		14		15		16	
S_1	2.55	0.121	2.47	0.109	2.53	0.107	2.49	0.123	2.51	0.091	2.58	0.078	2.51	0.071	2.58	0.065
S_2	3.02	0.027	3.47	0.004	3.21	0.03	3.32	0.025	3.04	0.028	3.11	0.01	3.15	0.008	3.08	0.005
S_3	3.46	0.094	3.54	0.096	3.57	0.132	3.53	0.086	3.48	0.04	3.74	0.084	3.76	0.229	3.71	0.023
S_4	3.78	0.153	3.7	0.075	3.65	0.029	3.71	0.097	3.72	0.289	3.84	0.069	3.82	0.033	3.77	0.192
S_5	4.07	0.041	3.93	0.071	3.94	0.21	3.92	0.153	3.9	0.022	4.23	0.026	4.16	0.079	4.26	0.03
S_6	4.28	0.078	4.02	0.197	4.1	0.026	4.07	0.062	4.29	0.062	4.32	0.123	4.37	0.029	4.35	0.038
S_7	4.51	0.141	4.67	0.249	4.62	0.287	4.63	0.208	4.63	0.199	4.54	0.022	4.56	0.016	4.54	0.011
S_8	4.75	0.048	4.79	0.028	4.79	0.031	4.83	0.029	4.71	0.008	4.58	0.059	4.61	0.105	4.62	0.188
S_9	4.82	0.02	4.84	0.001	4.85	0.025	4.97	0.002	4.75	0.016	4.65	0.102	4.82	0.033	4.65	0.002
S_{10}	4.94	0.007	5.01	0.064	5.01	0.012	5.04	0.024	4.83	0.012	5.01	0.002	4.92	0.018	4.7	0.028

2.2 BZ-azo-N,N-DMT

(17)

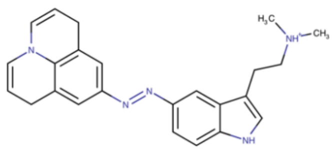

(18)

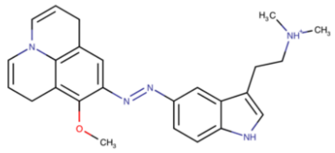

(19)

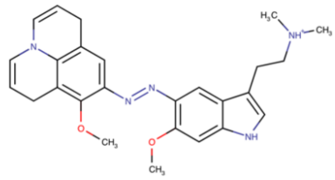

(20)

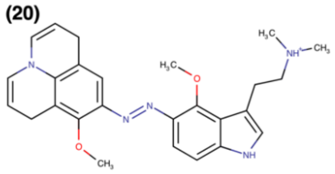

(21)

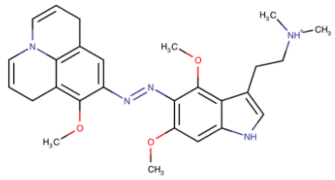

(22)

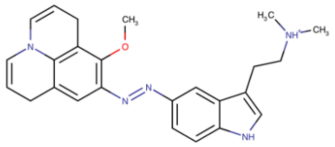

(23)

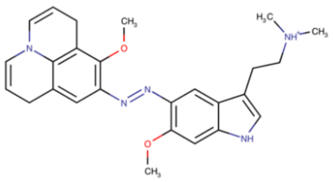

(24)

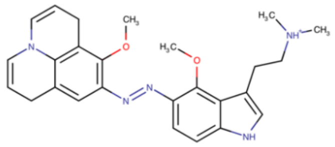

(25)

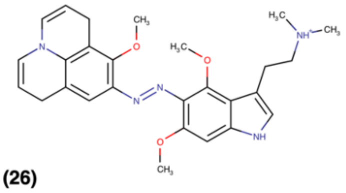

(26)

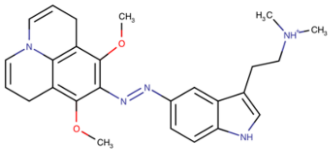

(27)

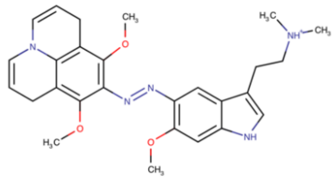

(28)

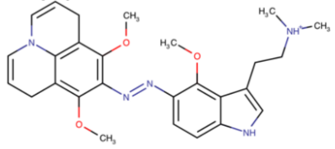

(29)

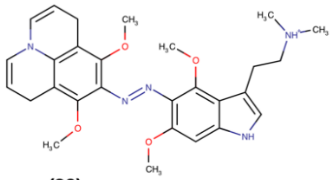

(30)

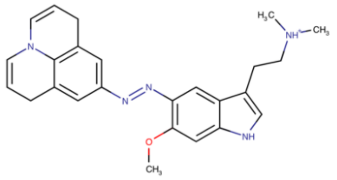

(31)

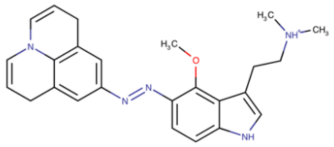

(32)

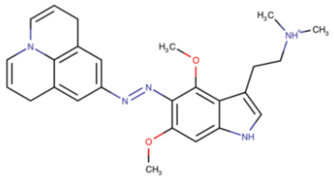

Figure S2: Chemical structure of the BZ-azo-N,N-DMT **(17)** to **(32)** in the trans isomer.

Table S2: Energies and oscillator strengths for the first 10 singlet excited states for the BZ-azo-N,N-DMT **(17)** to **(32)** compounds.

Trans																
	E(eV)	f	E(eV)	f	E(eV)	f	E(eV)	f	E(eV)	f	E(eV)	f	E(eV)	f	E(eV)	f
	17		18		19		20		21		22		23		24	
S_1	2.23	0.453	2.23	0.479	2.28	0.482	2.17	0.482	2.23	0.464	2.31	0.493	2.37	0.482	2.25	0.489
S_2	2.67	0	2.63	0	2.5	0	2.58	0	2.44	0.002	2.56	0	2.43	0.013	2.54	0
S_3	3.3	0.087	3.28	0.119	3.06	0.032	3.07	0.101	2.92	0.025	3.34	0.108	3.11	0.037	3.12	0.093
S_4	3.54	0.648	3.43	0.417	3.44	0.425	3.38	0.416	3.36	0.424	3.52	0.428	3.57	0.536	3.48	0.417
S_5	3.62	0.014	3.61	0.162	3.65	0.24	3.56	0.114	3.58	0.191	3.66	0.135	3.68	0.105	3.62	0.107
S_6	3.76	0.022	3.75	0.032	3.84	0.06	3.79	0.043	3.83	0.052	3.78	0.042	3.89	0.007	3.83	0.057
S_7	3.91	0.023	3.87	0.012	3.93	0.006	3.86	0.006	3.94	0.005	3.86	0.041	3.93	0.108	3.85	0.006
S_8	4.21	0.018	4.18	0.014	4.27	0.004	4.16	0.011	4.25	0.002	4.23	0.02	4.3	0.006	4.2	0.015
S_9	4.57	0.145	4.41	0.008	4.47	0.018	4.36	0.008	4.42	0.014	4.48	0	4.38	0.002	4.48	0.002
S_{10}	4.64	0.012	4.6	0.012	4.51	0.005	4.54	0.05	4.53	0.005	4.56	0.007	4.56	0	4.53	0.158
Cis																
	E(eV)	f	E(eV)	f	E(eV)	f	E(eV)	f	E(eV)	f	E(eV)	f	E(eV)	f	E(eV)	f
	17		18		19		20		21		22		23		24	
S_1	2.26	0.08	2.22	0.148	2.29	0.135	2.25	0.144	2.27	0.125	2.22	0.147	2.25	0.149	2.25	0.144
S_2	2.85	0.02	2.7	0.019	2.74	0.017	2.68	0.026	2.75	0.02	2.7	0.019	2.69	0.018	2.68	0.026
S_3	3.38	0.011	3.16	0.005	2.95	0.008	3.11	0.003	2.93	0.01	3.16	0.005	2.91	0.009	3.11	0.003
S_4	3.49	0.04	3.52	0.014	3.55	0.019	3.53	0.001	3.45	0.003	3.52	0.015	3.53	0.02	3.53	0.001
S_5	3.53	0.022	3.64	0.017	3.59	0.011	3.66	0.011	3.63	0.015	3.64	0.018	3.63	0.006	3.66	0.011
S_6	3.82	0.062	3.68	0.025	3.74	0.016	3.73	0.069	3.75	0.073	3.68	0.024	3.71	0.023	3.73	0.069
S_7	3.95	0.019	3.81	0.093	3.93	0.052	3.88	0.043	3.84	0.033	3.81	0.094	3.82	0.071	3.88	0.043
S_8	4.1	0.033	4.08	0.009	4.11	0.013	4.14	0.012	4.03	0.018	4.07	0.009	4.14	0.013	4.14	0.012
S_9	4.34	0.009	4.3	0.017	4.3	0.134	4.28	0.043	4.31	0.085	4.3	0.017	4.3	0.106	4.28	0.043
S_{10}	4.43	0.11	4.38	0.088	4.45	0.01	4.34	0.082	4.45	0.062	4.38	0.089	4.44	0.038	4.34	0.082

Trans																
	E(eV)	f	E(eV)	f	E(eV)	f	E(eV)	f	E(eV)	f	E(eV)	f	E(eV)	f	E(eV)	f
	25		26		27		28		29		30		31		32	
S_1	2.31	0.481	2.28	0.461	2.35	0.51	2.24	0.504	2.3	0.374	2.29	0.449	2.2	0.458	2.24	0.438
S_2	2.38	0.003	2.59	0.017	2.38	0.001	2.49	0.008	2.32	0.132	2.54	0	2.63	0	2.47	0.001
S_3	2.95	0.03	3.35	0.101	3.04	0.028	3.12	0.098	2.94	0.026	3.02	0.034	3.16	0.149	2.93	0.029
S_4	3.49	0.554	3.49	0.058	3.49	0.206	3.43	0.2	3.45	0.547	3.59	0.682	3.52	0.538	3.5	0.667
S_5	3.65	0.057	3.56	0.512	3.56	0.396	3.48	0.296	3.51	0.035	3.64	0.042	3.61	0.01	3.62	0.02
S_6	3.86	0.038	3.75	0.022	3.86	0.055	3.83	0.023	3.92	0.017	3.88	0.054	3.82	0.023	3.92	0.061
S_7	3.97	0.048	3.91	0.017	3.94	0.012	3.91	0.014	3.96	0.036	3.97	0.017	3.89	0.024	3.94	0.002
S_8	4.29	0.006	4.12	0.007	4.26	0.003	4.15	0.01	4.26	0.005	4.28	0.005	4.19	0.016	4.27	0.003
S_9	4.46	0.001	4.37	0.001	4.29	0.006	4.34	0.001	4.32	0.002	4.54	0	4.54	0.138	4.55	0.13
S_{10}	4.55	0.002	4.47	0.007	4.42	0.009	4.48	0.068	4.4	0.002	4.59	0.238	4.57	0.005	4.58	0.046
Cis																
	E(eV)	f	E(eV)	f	E(eV)	f	E(eV)	f	E(eV)	f	E(eV)	f	E(eV)	f	E(eV)	f
	25		26		27		28		29		30		31		32	
S_1	2.27	0.125	2.2	0.131	2.25	0.144	2.23	0.115	2.27	0.126	2.31	0.138	2.26	0.136	2.29	0.138
S_2	2.75	0.02	2.78	0.016	2.76	0.018	2.79	0.023	2.79	0.02	2.71	0.017	2.69	0.023	2.72	0.019
S_3	2.93	0.01	3.2	0.007	2.9	0.018	3.13	0.006	2.87	0.014	2.95	0.007	3.11	0.004	2.87	0.009
S_4	3.45	0.003	3.53	0.007	3.51	0.08	3.57	0.008	3.51	0.053	3.55	0.033	3.56	0.024	3.55	0.031
S_5	3.63	0.015	3.58	0.107	3.58	0.002	3.62	0.071	3.6	0.002	3.6	0.005	3.71	0.027	3.63	0.007
S_6	3.75	0.073	3.68	0.045	3.73	0.034	3.71	0.017	3.8	0.027	3.75	0.018	3.79	0.008	3.86	0.018
S_7	3.84	0.033	3.81	0.035	3.81	0.029	3.83	0.034	3.84	0.03	4.14	0.014	4.03	0.026	4.08	0.022
S_8	4.03	0.018	4	0.006	4.12	0.007	3.97	0.009	4.06	0.006	4.18	0.01	4.12	0.013	4.16	0.009
S_9	4.31	0.085	4.22	0.05	4.26	0.069	4.15	0.088	4.19	0.089	4.32	0.13	4.3	0.024	4.35	0.106
S_{10}	4.45	0.062	4.43	0.062	4.42	0.097	4.47	0.09	4.53	0.104	4.4	0.007	4.37	0.188	4.46	0.008

2.3 PQ-azo-N,N-DMT

(33)

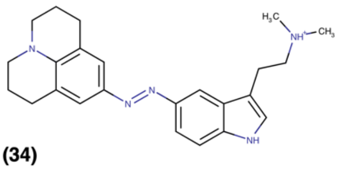

(34)

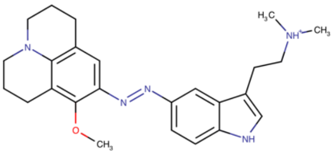

(35)

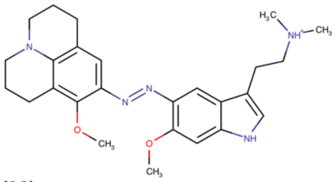

(36)

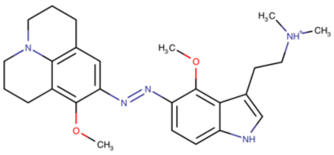

(37)

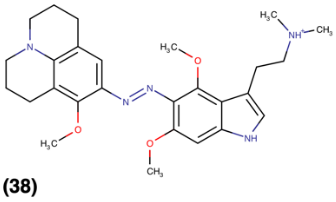

(38)

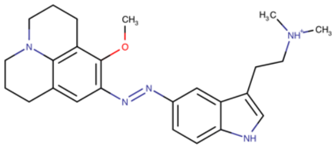

(39)

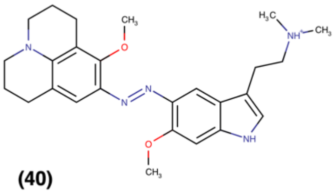

(40)

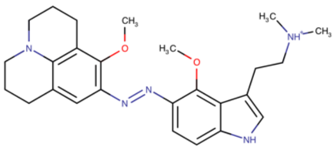

(41)

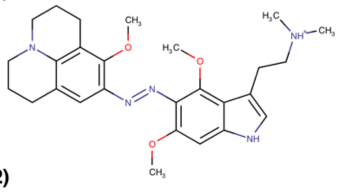

(42)

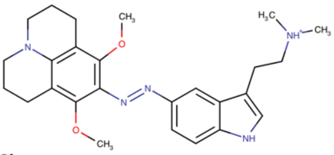

(43)

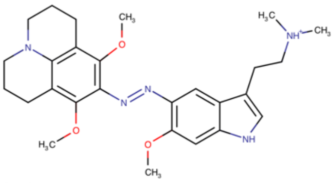

(44)

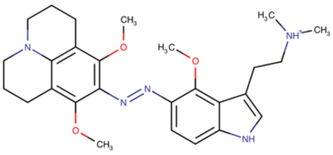

(45)

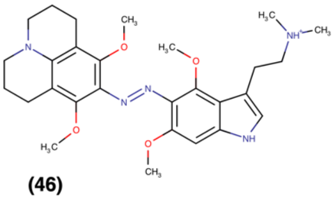

(46)

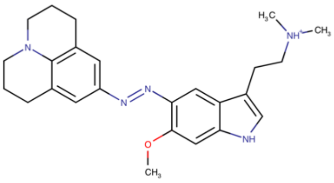

(47)

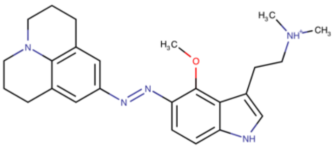

(48)

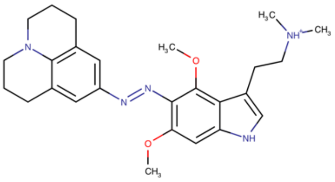

Figure S3: Chemical structure of the PQ-azo-N,N-DMT **(33)** to **(48)** in the trans isomer.

Table S3: Energies and oscillator strengths for the first 10 singlet excited states for the PQ-azo-N,N-DMT **(33)** to **(48)** compounds.

Trans																
	E(eV)	f	E(eV)	f	E(eV)	f	E(eV)	f	E(eV)	f	E(eV)	f	E(eV)	f	E(eV)	f
	33		34		35		36		37		38		39		40	
S_1	2.64	0.688	2.68	0.025	2.55	0.003	2.63	0.09	2.5	0.001	2.62	0.002	2.48	0.003	2.56	0.04
S_2	2.76	0.401	2.8	1.22	2.8	1.178	2.75	1.116	2.78	1.12	2.9	1.287	2.89	1.215	2.85	1.275
S_3	3.25	0.018	3.49	0.009	3.21	0.048	3.34	0.014	3.14	0.037	3.49	0.007	3.28	0.06	3.44	0.011
S_4	3.81	0.014	3.73	0.011	3.78	0.011	3.69	0.013	3.75	0.011	3.8	0.006	3.85	0.003	3.87	0.004
S_5	3.9	0.122	3.92	0.051	3.98	0.039	3.94	0.054	4	0.049	3.94	0.042	4.01	0.05	3.94	0.051
S_6	3.95	0.014	4.03	0.08	4.13	0.147	4	0.058	4.14	0.128	4.09	0.07	4.18	0.111	4.09	0.042
S_7	4.25	0.128	4.32	0.094	4.32	0.06	4.3	0.096	4.31	0.058	4.33	0.002	4.26	0.004	4.33	0.122
S_8	4.6	0.011	4.49	0.001	4.4	0.001	4.53	0.004	4.45	0.014	4.35	0.113	4.33	0.092	4.36	0.002
S_9	4.7	0.205	4.59	0.016	4.64	0.004	4.54	0.016	4.63	0.008	4.7	0.019	4.65	0	4.65	0.002
S_{10}	5.02	0.013	4.81	0.232	4.71	0.306	4.74	0.247	4.74	0.281	4.75	0.245	4.72	0.327	4.77	0.268
Cis																
	E(eV)	f	E(eV)	f	E(eV)	f	E(eV)	f	E(eV)	f	E(eV)	f	E(eV)	f	E(eV)	f
	33		34		35		36		37		38		39		40	
S_1	2.51	0.141	2.27	0.075	2.37	0.094	2.36	0.127	2.43	0.132	2.39	0.152	2.42	0.159	2.43	0.129
S_2	3.32	0.159	3.19	0.018	3.12	0.014	3.15	0.056	3.16	0.015	3.26	0.084	3.11	0.009	3.15	0.108
S_3	3.44	0.026	3.45	0.007	3.3	0.049	3.4	0.01	3.19	0.075	3.38	0.013	3.31	0.108	3.28	0.012
S_4	3.78	0.081	3.62	0.081	3.69	0.003	3.63	0.044	3.72	0.004	3.68	0.104	3.67	0.038	3.54	0.005
S_5	3.86	0.023	3.73	0.003	3.81	0.157	3.72	0.015	3.79	0.111	3.78	0.014	3.87	0.09	3.79	0.033
S_6	3.97	0.01	3.99	0.133	3.92	0.087	3.99	0.104	3.9	0.06	3.94	0.036	3.92	0.016	3.89	0.07
S_7	4.28	0.078	4.01	0.078	4.14	0.03	4.08	0.033	4.16	0.038	4.16	0.105	4.16	0.097	4.18	0.077
S_8	4.46	0.02	4.56	0.06	4.55	0.161	4.43	0.066	4.54	0.086	4.51	0.025	4.47	0.156	4.43	0.07
S_9	4.54	0.12	4.63	0.128	4.7	0.053	4.67	0.111	4.57	0.015	4.6	0.12	4.63	0.014	4.59	0.033
S_{10}	4.8	0.047	4.71	0.089	4.72	0.034	4.71	0.015	4.59	0.092	4.72	0.009	4.72	0.008	4.68	0.085

Trans																
	E(eV)	f	E(eV)	f	E(eV)	f	E(eV)	f	E(eV)	f	E(eV)	f	E(eV)	f	E(eV)	f
	41		42		43		44		45		46		47		48	
S_1	2.4	0.007	2.54	0.003	2.45	0.077	2.49	0.042	2.32	0.018	2.6	0.005	2.67	0.164	2.52	0.004
S_2	2.86	1.206	2.89	1.284	2.93	1.026	2.85	1.199	2.87	1.174	2.8	1.18	2.76	1.057	2.77	1.15
S_3	3.2	0.046	3.46	0.008	3.29	0.066	3.39	0.012	3.19	0.054	3.22	0.045	3.35	0.014	3.15	0.038
S_4	3.82	0.005	3.64	0.014	3.71	0.01	3.61	0.014	3.66	0.013	3.92	0.019	3.88	0.006	3.9	0.009
S_5	4.04	0.067	3.95	0.042	3.96	0.038	3.99	0.046	4.02	0.05	4	0.034	3.95	0.054	4.01	0.069
S_6	4.18	0.077	4.05	0.052	4.14	0.08	4.02	0.042	4.14	0.043	4.13	0.167	4.01	0.078	4.13	0.142
S_7	4.31	0.002	4.27	0.001	4.34	0.029	4.35	0.003	4.29	0.032	4.32	0.078	4.28	0.125	4.3	0.082
S_8	4.32	0.114	4.41	0.079	4.35	0.079	4.39	0.082	4.37	0.076	4.45	0	4.59	0	4.47	0.007
S_9	4.6	0.003	4.55	0.014	4.62	0.001	4.52	0.014	4.58	0.002	4.72	0.308	4.76	0.253	4.74	0.005
S_{10}	4.73	0.029	4.71	0.154	4.71	0.304	4.71	0.063	4.66	0.009	4.82	0	5.01	0	4.75	0.293
Cis																
	E(eV)	f	E(eV)	f	E(eV)	f	E(eV)	f	E(eV)	f	E(eV)	f	E(eV)	f	E(eV)	f
	41		42		43		44		45		46		47		48	
S_1	2.46	0.135	2.32	0.06	2.34	0.07	2.49	0.153	2.5	0.096	2.51	0.127	2.52	0.184	2.51	0.139
S_2	3.02	0.01	3.24	0.011	3.11	0.019	3.26	0.146	3.04	0.004	3.11	0.015	3.21	0.161	3.04	0.022
S_3	3.16	0.11	3.42	0.013	3.29	0.028	3.37	0.021	3.27	0.174	3.33	0.143	3.41	0.142	3.26	0.264
S_4	3.6	0.024	3.56	0.002	3.59	0.002	3.74	0.052	3.62	0.059	3.68	0.023	3.75	0.057	3.67	0.034
S_5	3.86	0.013	3.76	0.065	3.83	0.176	3.81	0.045	3.77	0.003	3.87	0.044	3.82	0.009	3.87	0.044
S_6	4.01	0.044	3.97	0.206	3.93	0.071	3.88	0.027	4.05	0.047	3.94	0.05	4.02	0.002	4.1	0.036
S_7	4.17	0.088	4.14	0.037	4.14	0.038	4.22	0.066	4.22	0.079	4.3	0.05	4.32	0.097	4.34	0.06
S_8	4.52	0.088	4.59	0.19	4.58	0.191	4.43	0.041	4.48	0.116	4.38	0.126	4.41	0.061	4.41	0.119
S_9	4.58	0.03	4.61	0.004	4.66	0.074	4.65	0.147	4.6	0.03	4.57	0.019	4.68	0.042	4.51	0.036
S_{10}	4.62	0.065	4.68	0.016	4.69	0.06	4.73	0.002	4.79	0.02	4.6	0.032	4.78	0.031	4.78	0.013

2.4 NH2-azo-N,N-DMT

(49)

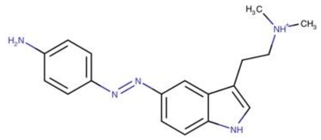

(50)

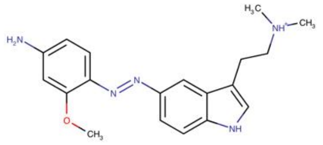

(51)

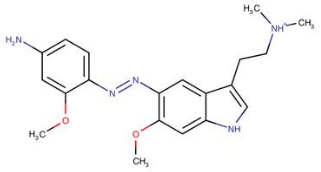

(52)

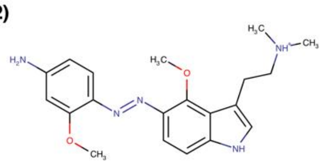

(53)

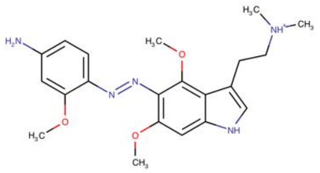

(54)

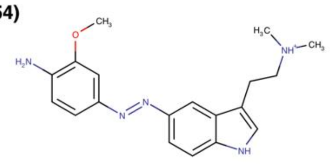

(55)

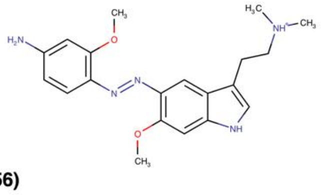

(56)

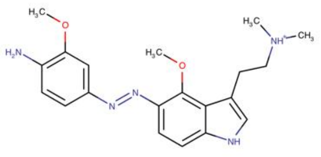

(57)

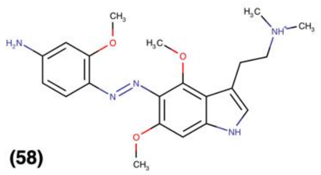

(58)

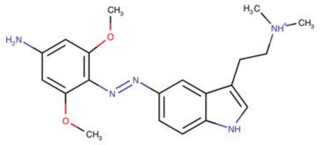

(59)

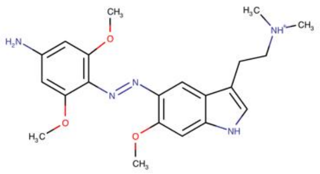

(60)

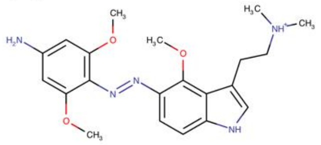

(61)

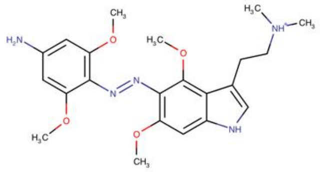

(62)

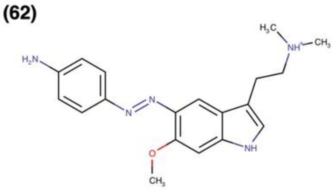

(63)

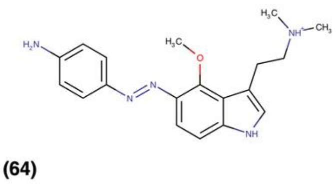

(64)

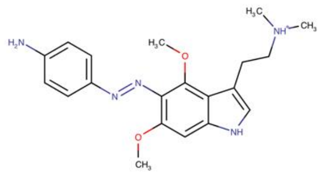

Figure S4: Chemical structure of the NH2-azo-N,N-DMT **(49)** to **(64)** in the trans isomer.

Table S4: Energies and oscillator strengths for the first 10 singlet excited states for the NH2-azo-N.N-DMT **(49)** to **(64)** compounds.

Trans																
	E(eV)	f	E(eV)	f	E(eV)	f	E(eV)	f	E(eV)	f	E(eV)	f	E(eV)	f	E(eV)	f
	49		50		51		52		53		54		55		56	
S_1	2.68	0.286	2.71	0.002	2.6	0.229	2.67	0.003	2.49	0.106	2.69	0	2.57	0.072	2.63	0.001
S_2	2.81	0.861	3.04	1.191	2.94	0.64	2.95	1.116	3.04	0.522	3.18	1.289	3.01	0.853	3.09	1.104
S_3	3.23	0.039	3.44	0.009	3.07	0.139	3.32	0.039	3.12	0.492	3.64	0.009	3.2	0.17	3.48	0.103
S_4	3.96	0.052	3.83	0.032	3.81	0.023	3.78	0.027	3.86	0.025	3.97	0.038	3.9	0.022	3.94	0.048
S_5	4.06	0.08	4.11	0.058	3.98	0.002	4.17	0.067	4.14	0.006	4.13	0.062	4.1	0.001	4.23	0.066
S_6	4.13	0.019	4.24	0.008	4.31	0.054	4.18	0.002	4.29	0.076	4.32	0	4.38	0.104	4.32	0.006
S_7	4.46	0.116	4.49	0	4.65	0.128	4.58	0.001	4.61	0.011	4.33	0.004	4.59	0.053	4.44	0.002
S_8	4.64	0.018	4.74	0.076	4.7	0.153	4.7	0.081	4.65	0.034	4.71	0.125	4.62	0.089	4.71	0.132
S_9	4.72	0.181	4.81	0.315	4.78	0.053	4.79	0.274	4.72	0.262	4.87	0.261	4.83	0.225	4.78	0.047
S_{10}	5.04	0	5.15	0	4.93	0.007	5.04	0.057	4.95	0.009	4.95	0	4.94	0.007	4.92	0.068
Cis																
	E(eV)	f	E(eV)	f	E(eV)	f	E(eV)	f	E(eV)	f	E(eV)	f	E(eV)	f	E(eV)	f
	49		50		51		52		53		54		55		56	
S_1	2.53	0.127	2.49	0.128	2.53	0.12	2.53	0.089	2.55	0.118	2.51	0.162	2.56	0.131	2.48	0.179
S_2	3.36	0.055	3.45	0.028	3.25	0.033	3.25	0.018	3.12	0.026	3.46	0.083	3.25	0.04	3.33	0.009
S_3	3.49	0.178	3.64	0.179	3.52	0.111	3.51	0.091	3.49	0.091	3.62	0.146	3.45	0.106	3.55	0.35
S_4	3.88	0.049	3.85	0.087	3.8	0.257	3.79	0.162	3.71	0.275	3.84	0.112	3.88	0.104	3.81	0.029
S_5	3.9	0.086	3.87	0.136	4.03	0.004	4.01	0.004	4	0.007	3.88	0.026	3.93	0.005	3.87	0.037
S_6	4.12	0.018	4.01	0.017	4.08	0.043	4.16	0.09	4.18	0.054	4.02	0.049	4.07	0.103	3.98	0.05
S_7	4.55	0.052	4.5	0.075	4.51	0.11	4.51	0.051	4.55	0.106	4.55	0.042	4.51	0.16	4.53	0.028
S_8	4.56	0.016	4.63	0.08	4.63	0.125	4.61	0.06	4.63	0.088	4.62	0.127	4.64	0.059	4.57	0.185
S_9	4.63	0.134	4.9	0.053	4.79	0.066	4.83	0.06	4.73	0.031	4.83	0.1	4.7	0.066	4.8	0.039
S_{10}	4.86	0.019	5.05	0.04	4.98	0.022	4.95	0.004	4.94	0.005	5.09	0.053	5.12	0.034	5.05	0.048

Trans																
	E(eV)	f	E(eV)	f	E(eV)	f	E(eV)	f	E(eV)	f	E(eV)	f	E(eV)	f	E(eV)	f
	57		58		59		60		61		62		63		64	
S_1	2.52	0.067	2.53	0.013	2.46	0.052	2.55	0.007	2.43	0.008	2.63	0	2.75	0.037	2.53	0.257
S_2	3.16	0.717	3.07	0.946	2.99	0.828	3.1	1.151	3.12	0.762	3.08	0.898	3.01	0.981	2.86	0.838
S_3	3.28	0.398	3.44	0.085	3.19	0.152	3.46	0.067	3.21	0.367	3.28	0.313	3.4	0.13	3.1	0.038
S_4	4.02	0.032	3.62	0.038	3.61	0.035	3.66	0.032	3.81	0.037	4.2	0.006	4.22	0.059	3.89	0.009
S_5	4.18	0.035	4.18	0.122	4.12	0.009	4.16	0.061	4.26	0.006	4.29	0.024	4.23	0.014	3.97	0.067
S_6	4.3	0.027	4.27	0.066	4.36	0.077	4.31	0.013	4.3	0.016	4.38	0.076	4.32	0.019	4.13	0.117
S_7	4.49	0.028	4.31	0.027	4.51	0.09	4.34	0.018	4.42	0.067	4.5	0	4.68	0.1	4.29	0.087
S_8	4.59	0.079	4.81	0.081	4.79	0.134	4.78	0.193	4.71	0.021	4.74	0.054	4.7	0.007	4.57	0.004
S_9	4.74	0.235	4.88	0.058	4.82	0.078	4.89	0.093	4.74	0.26	4.75	0.091	4.77	0.132	4.69	0.264
S_{10}	4.83	0.053	4.97	0.042	4.95	0.041	5.01	0.029	4.76	0.01	4.77	0.301	4.9	0.003	4.79	0.004
Cis																
	E(eV)	f	E(eV)	f	E(eV)	f	E(eV)	f	E(eV)	f	E(eV)	f	E(eV)	f	E(eV)	f
	57		58		59		60		61		62		63		64	
S_1	2.52	0.142	2.4	0.135	2.48	0.128	2.44	0.151	2.5	0.122	2.55	0.091	2.51	0.08	2.53	0.078
S_2	3.08	0.032	3.45	0.049	3.25	0.037	3.36	0.014	3.11	0.033	3.18	0.008	3.21	0.008	3.11	0.007
S_3	3.43	0.145	3.54	0.103	3.5	0.145	3.53	0.17	3.46	0.079	3.63	0.104	3.58	0.206	3.59	0.222
S_4	3.78	0.107	3.64	0.026	3.63	0.064	3.63	0.053	3.66	0.269	3.77	0.041	3.84	0.04	3.75	0.034
S_5	3.9	0.007	3.77	0.083	3.92	0.105	3.89	0.086	3.86	0.014	4.15	0.041	4.08	0.081	4.15	0.068
S_6	4.12	0.095	4.04	0.193	3.97	0.081	3.95	0.107	4.15	0.046	4.17	0.1	4.29	0.015	4.29	0.02
S_7	4.51	0.129	4.65	0.169	4.59	0.247	4.61	0.169	4.6	0.14	4.55	0.083	4.58	0.031	4.56	0.023
S_8	4.65	0.085	4.68	0.042	4.69	0.036	4.71	0.039	4.65	0.087	4.6	0.054	4.6	0.093	4.6	0.146
S_9	4.72	0.027	4.78	0.012	4.76	0.034	4.8	0.016	4.72	0.003	4.7	0.063	4.82	0.072	4.68	0.002
S_{10}	4.99	0.004	4.81	0.003	4.85	0.004	4.93	0.006	4.76	0.033	4.89	0.013	4.88	0.019	4.76	0.042

2.5 NOCH3-azo-N,N-DMT

(65)

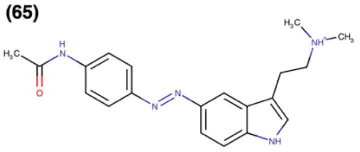

(73)

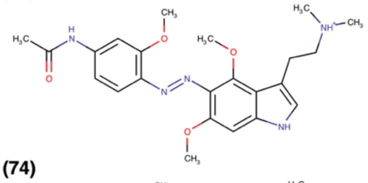

(66)

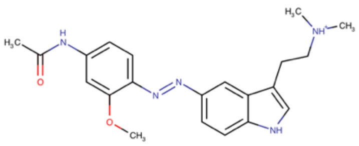

(74)

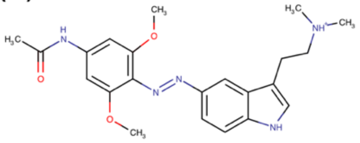

(67)

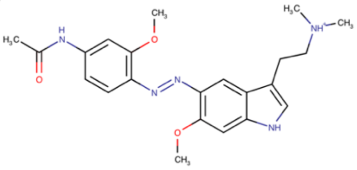

(75)

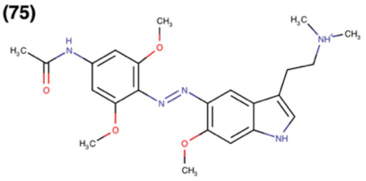

(68)

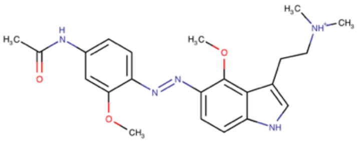

(76)

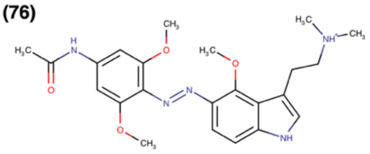

(69)

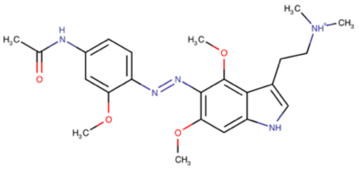

(77)

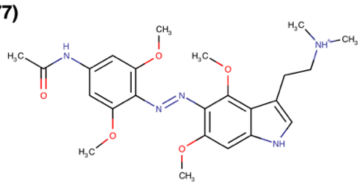

(70)

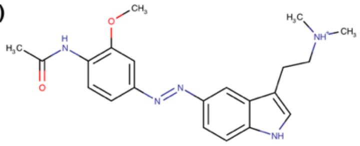

(78)

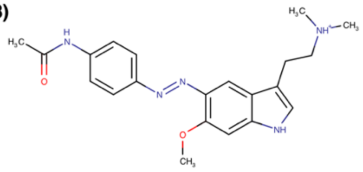

(71)

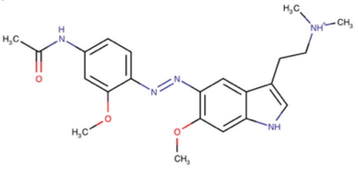

(79)

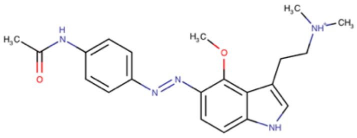

(72)

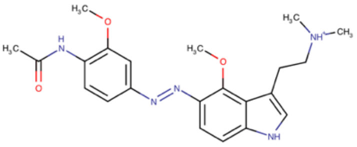

(80)

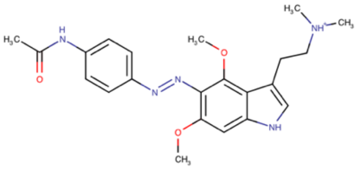

Figure S5: Chemical structure of the NOCH3-azo-N.N-DMT **(65)** to **(80)** in the trans isomer.

Table S5: Energies and oscillator strengths for the first 10 singlet excited states for the NOCH3-azo-N,N-DMT **(65)** to **(80)** compounds

Trans																
	E(eV)	f	E(eV)	f	E(eV)	f	E(eV)	f	E(eV)	f	E(eV)	f	E(eV)	f	E(eV)	f
	65		66		67		68		69		70		71		72	
S_1	2.68	0.002	2.62	0.002	2.52	0.169	2.6	0.072	2.41	0.065	2.58	0.001	2.47	0.047	2.6	0.001
S_2	3.15	1.256	3.04	1.235	2.83	0.151	2.94	0.858	2.92	0.13	3.17	1.278	2.91	0.354	3.01	0.968
S_3	3.33	0.076	3.3	0.018	2.97	0.732	3.19	0.259	3.06	0.991	3.46	0.032	3.1	0.747	3.42	0.298
S_4	4.14	0.013	3.75	0.082	3.7	0.046	3.74	0.071	3.77	0.063	3.87	0.126	3.77	0.06	3.92	0.113
S_5	4.23	0.002	4.19	0.017	4	0.041	4.16	0.018	4.15	0.009	4.3	0.035	4.13	0.036	4.29	0.03
S_6	4.31	0	4.34	0	4.3	0	4.35	0.001	4.39	0.012	4.39	0.052	4.4	0	4.45	0.043
S_7	4.47	0.058	4.35	0.062	4.44	0.072	4.37	0.05	4.41	0.054	4.43	0.006	4.47	0.097	4.52	0
S_8	4.69	0	4.58	0	4.65	0.155	4.66	0.049	4.65	0.119	4.47	0	4.64	0.099	4.56	0
S_9	4.85	0.402	4.78	0.243	4.74	0.009	4.75	0.006	4.67	0.092	4.79	0.254	4.71	0.15	4.7	0.121
S_{10}	4.9	0.04	4.95	0.117	4.81	0.006	4.8	0.184	4.79	0.05	4.87	0.007	4.77	0.023	4.77	0.059

Cis																
	E(eV)	f	E(eV)	f	E(eV)	f	E(eV)	f	E(eV)	f	E(eV)	f	E(eV)	f	E(eV)	f
	65		66		67		68		69		70		71		72	
S_1	2.59	0.06	2.43	0.067	2.43	0.067	2.44	0.072	2.42	0.065	2.53	0.049	2.45	0.069	2.55	0.053
S_2	3.17	0.005	3.16	0.01	3	0.011	3.01	0.02	2.91	0.02	3.24	0.005	2.84	0.018	3.09	0.01
S_3	3.78	0.088	3.59	0.046	3.56	0.117	3.52	0.081	3.46	0.048	3.57	0.021	3.54	0.139	3.57	0.037
S_4	3.92	0.058	3.73	0.208	3.62	0.093	3.71	0.174	3.6	0.183	3.87	0.167	3.73	0.058	3.79	0.093
S_5	4.19	0.171	4.02	0.006	4.04	0.007	4.04	0.029	4.03	0.052	4.12	0.015	3.97	0.004	3.97	0.024
S_6	4.24	0.036	4.21	0.22	4.23	0.238	4.24	0.18	4.27	0.147	4.2	0.165	4.21	0.189	4.2	0.101
S_7	4.44	0.017	4.42	0.02	4.41	0.025	4.41	0.001	4.41	0.007	4.38	0.052	4.24	0.044	4.36	0.045
S_8	4.55	0.022	4.55	0.118	4.5	0.028	4.52	0.054	4.47	0.012	4.55	0.052	4.48	0.038	4.49	0.071
S_9	4.63	0.148	4.6	0.017	4.58	0.092	4.6	0.012	4.55	0.05	4.66	0.066	4.57	0.019	4.6	0.049
S_{10}	4.68	0.009	4.8	0.043	4.62	0.003	4.64	0.028	4.61	0.024	4.79	0.124	4.62	0.006	4.71	0.017

Trans																
	E(eV)	f	E(eV)	f	E(eV)	f	E(eV)	f	E(eV)	f	E(eV)	f	E(eV)	f	E(eV)	f
	73		74		75		76		77		78		79		80	
S_1	2.4	0.033	2.44	0.032	2.37	0.04	2.46	0.042	2.35	0.004	2.54	0.001	2.66	0.06	2.48	0.046
S_2	3.02	0.133	3.05	0.842	2.9	0.519	3.07	1.028	3	0.142	2.98	0.239	2.99	0.59	2.91	0.064
S_3	3.2	1.101	3.3	0.263	3.06	0.546	3.32	0.203	3.15	1.052	3.24	1.09	3.23	0.661	3.17	1.188
S_4	3.88	0.103	3.48	0.052	3.47	0.046	3.52	0.042	3.7	0.069	4.2	0.007	4.15	0.005	4.15	0.003
S_5	4.26	0.03	4.21	0.003	4.11	0.031	4.26	0.05	4.24	0.017	4.28	0	4.2	0.002	4.23	0.001
S_6	4.35	0.033	4.28	0.141	4.38	0.062	4.29	0.023	4.35	0.011	4.39	0	4.27	0	4.35	0
S_7	4.48	0	4.44	0.055	4.41	0.001	4.48	0	4.5	0.006	4.54	0.021	4.45	0.058	4.5	0.048
S_8	4.6	0.041	4.44	0.048	4.53	0.125	4.49	0.028	4.55	0.078	4.58	0.078	4.68	0.044	4.66	0.107
S_9	4.62	0.099	4.77	0.046	4.67	0.096	4.7	0.146	4.65	0.116	4.7	0.29	4.78	0.048	4.68	0.133
S_{10}	4.69	0.118	4.87	0.026	4.84	0.029	4.85	0.017	4.67	0.003	4.74	0.001	4.82	0.142	4.73	0.013

Cis																
	E(eV)	f	E(eV)	f	E(eV)	f	E(eV)	f	E(eV)	f	E(eV)	f	E(eV)	f	E(eV)	f
	73		74		75		76		77		78		79		80	
S_1	2.45	0.069	2.61	0.04	2.48	0.069	2.46	0.075	2.4	0.063	2.49	0.082	2.64	0.042	2.48	0.098
S_2	2.91	0.018	3.21	0.003	2.87	0.021	3.06	0.02	2.87	0.022	2.88	0.006	3.04	0.775	2.88	0.025
S_3	3.44	0.095	3.56	0.043	3.52	0.151	3.56	0.131	3.48	0.048	3.58	0.036	3.22	0.481	3.62	0.038
S_4	3.69	0.104	3.82	0.078	3.6	0.023	3.6	0.036	3.6	0.179	3.85	0.274	4.1	0.006	3.66	0.337
S_5	4.12	0.027	3.9	0.027	3.93	0.022	3.79	0.125	3.83	0.058	4.16	0.029	4.17	0.005	4.18	0.039
S_6	4.34	0.11	4.17	0.182	4.24	0.141	4.2	0.146	4.26	0.128	4.31	0.118	4.25	0	4.36	0.112
S_7	4.47	0.006	4.43	0.012	4.25	0.082	4.38	0.004	4.38	0.006	4.38	0.05	4.44	0.069	4.43	0.048
S_8	4.48	0.025	4.56	0.063	4.49	0.038	4.49	0.078	4.41	0.015	4.46	0.071	4.75	0.074	4.44	0.013
S_9	4.61	0.111	4.59	0.072	4.65	0.013	4.62	0.02	4.51	0.013	4.52	0.001	4.8	0.054	4.62	0.024
S_{10}	4.65	0.015	4.8	0.036	4.68	0.07	4.71	0.004	4.56	0.081	4.63	0.054	4.84	0.096	4.74	0.017

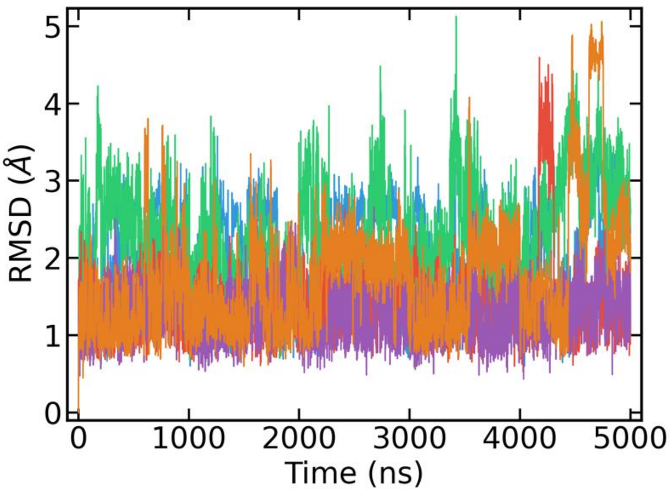

Figure S6: Root Mean Squared Deviation for LSD (purple), cis-**(1)** (orange), trans-**(1)** (green), cis-**(34)** (blue), and trans-**(34)** (red).

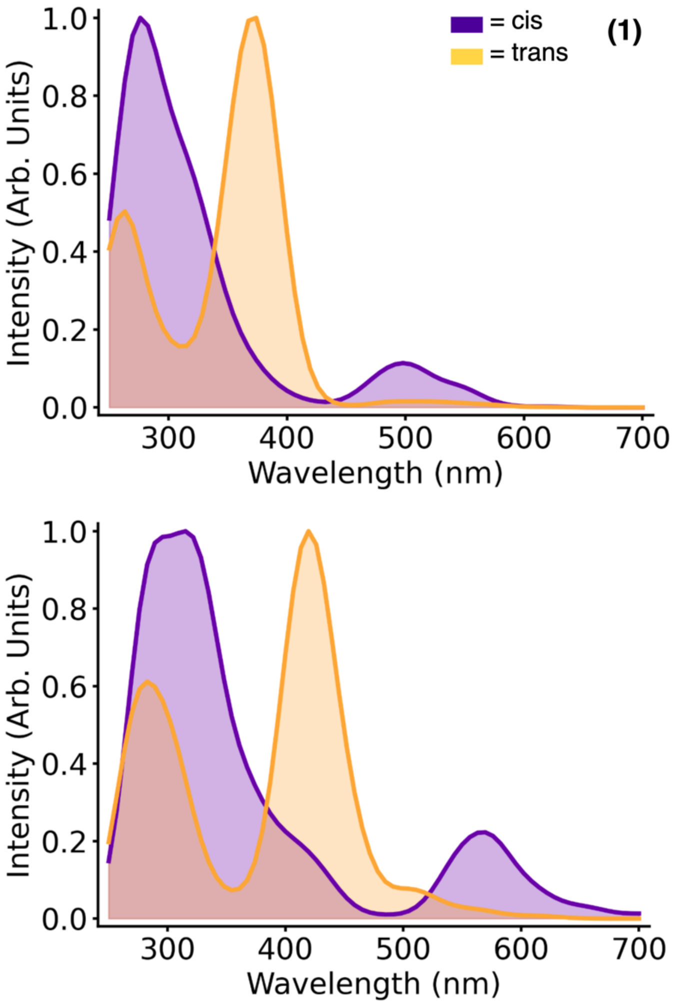

Figure S7: Absorption spectrum of the trans- and cis- azo-N,N-DMT (1) (top) and trans- and cis- PQ-azo-N,N-DMT (34) (bottom).
